# Long noncoding RNA expression profile in fibroblast-like synoviocytes from patients with rheumatoid arthritis

**DOI:** 10.1186/s13075-016-1129-4

**Published:** 2016-10-06

**Authors:** Yu Zhang, Yu-Zhong Xu, Ning Sun, Jian-Hong Liu, Fang-Fang Chen, Xiao-Long Guan, Ang Li, Fei Wang, Qin-Fei Zhao, Hai-Yong Wang, Shu-Sheng Song, Wei Yu, Jian-Ning Zhao, Xiao-Jun Li

**Affiliations:** 1 Department of Clinical Laboratory Science, Jinling Hospital, School of Medicine, Nanjing University, 305 East Zhongshan Road, Nanjing, 210002 China; 2 Department of Osteology, Jinling Hospital, School of Medicine, Nanjing University, 305 East Zhongshan Road, Nanjing, 210002 China; 3 State Key Laboratory of Analytical Chemistry for Life Science, Department of Chemistry, Nanjing University, Nanjing, 210093 China

**Keywords:** Rheumatoid arthritis, Fibroblast-like synoviocytes, Long noncoding RNA, Expression profile

## Abstract

**Background:**

Long noncoding RNAs (lncRNAs) have recently received wide attention as key molecules that mediate a variety of physiological and pathological processes by regulating gene expression; however, knowledge of lncRNAs in rheumatoid arthritis (RA) is limited. Thus, we investigated the lncRNA expression profile in fibroblast-like synoviocytes (FLSs) from patients with RA and explored the function of abundantly expressed lncRNAs.

**Methods:**

LncRNA and mRNA microarrays were performed to identify differentially expressed lncRNAs in RA FLSs compared with normal FLSs. Quantitative polymerase chain reaction (qPCR) was used to validate the results, and correlation analysis was used to analyze the relationship between these aberrantly expressed lncRNAs and clinical characteristics. A receiver operating characteristic (ROC) curve was constructed to evaluate the diagnostic value of the lncRNAs identified.

**Results:**

According to the gene expression profiles, 135 lncRNAs were differentially expressed between RA and normal FLSs. Furthermore, qPCR data showed that lncRNA ENST00000483588 was up-regulated and that three lncRNAs (ENST00000438399, uc004afb.1, and ENST00000452247) were down-regulated in RA FLSs. The expression level of ENST00000483588 was positively correlated with the level of C-reactive protein and the Simplified Disease Activity Index score. Moreover, the areas under the ROC curve were 0.85, 0.92, 0.97, and 0.92 for ENST00000483588, ENST00000438399, uc004afb.1, and ENST00000452247, respectively.

**Conclusions:**

The results indicate that the dysregulation of ENST00000483588, ENST00000438399, uc004afb.1, and ENST00000452247 may be involved in the pathological processes of RA and that these lncRNAs may have potential value for the diagnosis and assessment of the disease activity of RA.

**Electronic supplementary material:**

The online version of this article (doi:10.1186/s13075-016-1129-4) contains supplementary material, which is available to authorized users.

## Background

Rheumatoid arthritis (RA) is a systemic autoimmune disease characterized by chronic joint inflammation and variable degrees of bone and cartilage destruction. The thickened synovium in RA mainly includes two types of cells: macrophage-like synovial cells and fibroblast-like synovial cells (FLSs). As key effector cells in RA, FLSs have attracted increasing attention [1]. The mammalian genome encodes thousands of noncoding RNAs (ncRNAs).

Over the past few decades, most of the research attention in this field has been devoted to exploring the role of small ncRNAs such as microRNAs (~21–25 nucleotides), which regulate gene expression at the transcriptional and post-transcriptional levels. MicroRNA-124a was found to be a key regulator of the proliferation and secretion of monocyte chemoattractant protein 1 in RA FLSs [2]. In addition, microRNA-18a was reported to activate FLSs through a feedback loop in nuclear factor-kappa B signaling [3]. Moreover, a novel p53/microRNA-22/cyr61 axis in synovial cells was reported to play a role in regulating inflammation in RA [4]. Recently, long noncoding RNAs (lncRNAs), which had previously been thought to be nonfunctional RNAs, were shown to play important roles in chromatin remodeling, transcription control, post-transcriptional processing, and protein metabolism. LncRNAs have also been found to play important roles in many diseases such as cancer [5, 6], Alzheimer’s disease [7], cardiovascular disease [8], diabetes mellitus [9], and systemic lupus erythematosus [10]. However, knowledge of lncRNAs in RA FLSs remains limited. In this study, an lncRNA expression profile for RA was established, and the relationships between the expression levels of aberrantly expressed lncRNAs and clinical indices were analyzed. Moreover, we explored the value of these lncRNAs in diagnosing or assessing RA disease activity.

## Methods

### Patients and specimens

RA synovial tissue specimens were obtained from the knee joints of 10 patients with RA, who were undergoing total knee arthroplasty (two men and eight women, age range 38–65 years). In addition, normal synovial tissue specimens were obtained from the knee joints of 10 patients with trauma, who were undergoing arthroscopic surgery for knee ligament injury or meniscus injury (three men and seven women, age range 35–61 years). The clinical characteristics of the patients are shown in Additional file 1: Table S1. All patients with RA included in the study fulfilled the 2010 American College of Rheumatology/European league Against Rheumatism (ACR/EULAR) classification criteria for RA [11]. Written informed consent was obtained from each participant prior to sample collection. The study was approved by the Human Ethics Committee of Jinling Hospital.

### Isolation and culture of FLSs

Tissue specimens were minced into small pieces and digested for 2 hours with 2 mg/ml of type II collagenase (Invitrogen, Carlsbad, CA, USA) in high-glucose Dulbecco’s modified Eagle’s medium (DMEM) at 37 °C [12]. After centrifugation at 210 × *g* for 5 minutes, the precipitate was resuspended with 1 ml of high-glucose DMEM containing 10 % fetal bovine serum (FBS), 100 units/ml penicillin, and 100 units/ml streptomycin, and then cultured in 25-cm^2^ cell culture flasks (Corning) in a humidified 5 % CO_2_ incubator. After 10 hours, 4 ml of high-glucose DMEM containing 10 % FBS was added to the cell culture flask. All experiments were conducted using cells at passage 3.

### Flow cytometry

FLSs at passage 3 were identified by flow cytometry based on the expression of CD68 (a macrophage marker) and CD90 (a fibroblast marker) [13]. Cells were washed three times with phosphate-buffered saline (PBS) and were then incubated with fluorescein isothiocyanate (FITC)-conjugated anti-CD68 antibody, phycoerythrin (PE)-conjugated anti-CD90 antibody, FITC-conjugated mouse IgG2b, or PE-conjugated mouse IgG1 (Miltenyi Biotec, Germany) for 20 minutes in the dark. Cells were washed with PBS and then analyzed on a FACSCalibur flow cytometer (BD Biosciences, San Diego, CA, USA).

### Microarray analysis

Sample labeling and array hybridization were performed according to the Agilent One-Color Microarray-Based Gene Expression Analysis protocol (Agilent Technology). Briefly, RNA was purified using the RNeasy Mini Kit (Qiagen, Germany). Each sample was then amplified and labeled with cyanine-3-CTP. The labeled cRNAs were purified again with the RNeasy Mini Kit. The production of cRNAs needed to reach 1.65 μg to meet the requirements of the microarray. The specific activity of the labeled cRNAs needed to reach 9.0 pmol Cy3/μg cRNA. RNA quantity and quality were measured according to the A260 nm/A280 nm ratio using a NanoDrop ND-1000 spectrometer. RNA integrity was detected by standard denaturing agarose gel electrophoresis. For each microarray, 0.6 μg cRNA, 5 μl of 10× blocking agent, 1 μl of 25× fragmentation buffer, and nuclease-free water were added to reach a total volume of 25 μl: 25 μl of 2× GE Hybridization Buffer was then added to stop the fragmentation reaction. The hybridization solution and Arraystar Human LncRNA Microarray V3.0 were incubated at 65 °C for 17 hours in an Agilent Hybridization Oven. Approximately 30,586 lncRNAs and 26,109 coding transcripts can be detected using the third-generation lncRNA microarray. After washing the chip, a microarray scanner (Agilent DNA Microarray Scanner) was used to measure the fluorescence intensity. Agilent Feature Extraction Software was used to analyze the raw data.

### Volcano plots and hierarchical cluster analyses

The microarray data were log-transformed and normalized using quantile normalization. After filtering to remove unreliable transcripts, the remaining data were statistically analyzed to identify lncRNAs and mRNAs with significantly differential normalization. Volcano plots are useful tools for visualizing genes expressed differentially between two groups. Transcripts were distributed according to statistical significance (y-axis) and the magnitude of change (log2 ratio of RA FLSs/normal FLSs) (x-axis). Hierarchical cluster analysis was used to identify distinguishable RNA expression profiles between different samples.

### LncRNA classification

Analyzing the genomic context of lncRNAs can help to predict their functional roles. According to the sequence and relative position between lncRNAs and their associated protein-coding genes, the lncRNAs detected by microarray were characterized as natural antisense, intronic antisense, exon sense overlapping, intron sense overlapping, and bidirectional, and intergenic, among others [14]. Natural antisense lncRNAs are RNA molecules that are transcribed from the antisense strand and overlap with coding transcripts. Intronic antisense lncRNAs are RNA molecules that are transcribed from the antisense strand, but do not share overlapping exons. Exon sense overlapping lncRNAs can be considered as transcript variants of protein-coding mRNAs, as they overlap with coding transcripts from the same genomic strand. Intron sense overlapping lncRNAs overlap with the introns of annotated coding genes on the same genomic strand. A bidirectional lncRNA is oriented head-to-head with a protein-coding gene within 1000 bp. Intergenic lncRNAs involve no overlapping or bidirectional coding transcripts near the lncRNAs. The “others” category mainly included lncRNAs for which the associated genes were pseudogenes.

### Bioinformatics analysis

Pathway analysis and Gene Ontology (GO) analysis were applied to explore the potential roles that the differentially expressed mRNAs play in a biological pathway or GO function, including three categories: biological process, cellular component, and molecular function.

### Co-expression network

A gene co-expression network was built according to the normalized signal intensity of specifically expressed lncRNAs and mRNAs using the Cytoscape program. Pearson correlation analysis was used to evaluate the significance of the correlation between the expression levels between each pair of genes. Pearson correlation coefficients were selected for inclusion in the network when they were above 0.95. By analyzing the function of these mRNAs, we could link lncRNAs with particular functions and signaling pathways, which could facilitate the prediction of their biological functions and mechanisms of action. If the area of the node increased with increasing connections between mRNAs and lncRNAs, this indicated that the lncRNA may play an important role.

### Quantitative polymerase chain reaction (qPCR) analysis

The primers used for qPCR were synthesized by Kangcheng (Shanghai, China). Each PCR contained 2 μl template cDNA, 10 μl 2× SYBR Green mix (TaKaRa, Japan), 1 μl forward primer, 1 μl reverse primer, and nuclease-free water to a total volume of 20 μl. The PCR conditions were as follows: 95 °C for 10 minutes, followed by 40 cycles of 95 °C for 10 seconds and 60 °C for 60 seconds. *GAPDH* mRNA was used as an endogenous control to normalize lncRNA expression levels using the 2^-△△Ct^ method. The primer sequences used in this study were as follows: for *GAPDH*, 5′-GGGAAACTGTGGCGTGAT-3′ (forward) and 5′-GAGTGGGTGTCGCTGTTGA-3′ (reverse); for ENST00000483588, 5′-CACGTGAAAGGGGGAGAAA-3′ (forward) and 5′-CCAACAGCACAGAAGGCGT-3′ (reverse); for NR_073012, 5′-ATTCCCACGTATGCGGAGTG3′ (forward) and 5′-ACGGGTGTAGTAGGCGTTTC-3′ (reverse); for ENST00000567753, 5′-CGTGGCAGGACTTTGCTTTC-3′ (forward) and 5′-GGGGTCTTACTGTGTGGGGT-3′ (reverse); for uc003xhp.3, 5′-AGGTATCAGTCTGGGGGACC-3′ (forward) and 5′- ACGCACAGTGGAGGAATGAG-3′ (reverse); for ENST00000557804, 5′- CAGAACGATCAAGCGACCTC-3′ (forward) and 5′- CGTGGAAGGAAGATGACCC-3′ (reverse); for ENST00000438399, 5′- AGAAAGTCAAGGGAAGATAAGG-3′ (forward) and 5′- TAGTCAACCAGGGAAGCAGT-3′ (reverse); for uc004afb.1, 5′- AAACTATGCCTGATTTGTGGTC-3′ (forward) and 5′-CTGGTGTAAGAGCATGGGGT-3′ (reverse); for ENST00000412143, 5′- ATTACTCTTTCCCAGCCCAGC-3′ (forward) and 5′- GCCCTCCTTGCAGCATCAT-3′ (reverse); for ENST00000452247, 5′- GACTCCTCCTCCTGCTTTCAC-3′ (forward) and 5′-GGCAATAGAGCTGGATCTTGT-3′ (reverse).

### Statistical analysis

SPSS software 19.0 and GraphPad Prism 6.0 were used to analyze the data. The two-tailed Student *t* test and rank-sum test were used as appropriate to analyze the expression levels between two groups. The relationships between the expression levels of lncRNAs and clinical characteristics were analyzed by Pearson’s correlation coefficient. *P* values below 0.05 were regarded as statistically significant. The diagnostic value was evaluated with a receiver operating characteristic (ROC) curve.

## Results

### Identification of FLSs

**Fig. 1:**
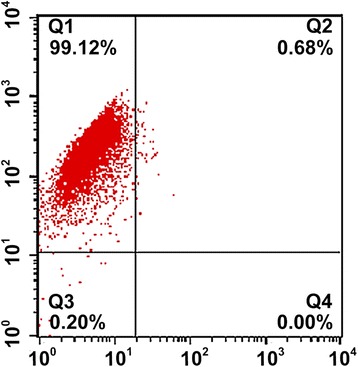
Identification of fibroblast-like synoviocytes (FLSs) by flow cytometry

The purity of FLSs at passage 3 was determined by flow cytometry. The purity of the third generation of FLSs of the primary culture reached 99.0 % (Fig. 1).

### LncRNA and mRNA expression profile in RA FLSs

We performed genome-wide analysis of the expression profiles of lncRNAs and mRNAs in three pairs of FLSs samples from patients with RA and patients with trauma using Arraystar Human LncRNA Microarray V3.0. Based on the criteria of a fold change >2.0 and a *P* value <0.05, 135 lncRNAs and 103 mRNAs were differentially expressed between the RA FLSs and normal FLSs (Additional files 2 and 3: Tables S2 and S3). Of the 135 lncRNAs identified, 62 lncRNAs were up-regulated and 73 lncRNAs were down-regulated in the RA FLSs. Of the 103 mRNAs detected, 36 mRNAs were up-regulated and 67 mRNAs were down-regulated in the RA FLSs.

### Volcano plots and hierarchical cluster

**Fig. 2:**
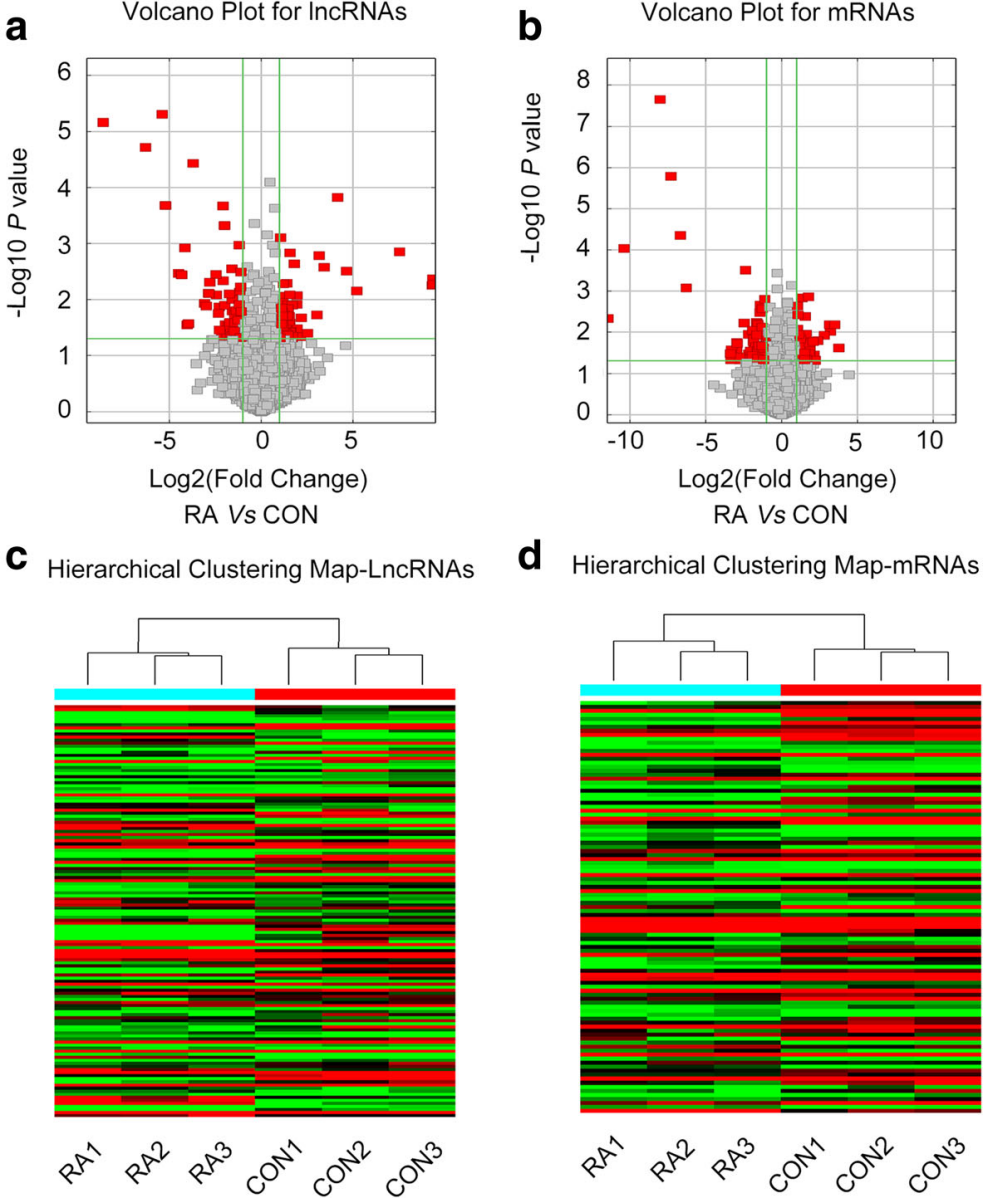
Volcano plot and heat map analyses of rheumatoid arthritis (*RA*) fibroblast-like synoviocyte (FLS) samples and normal FLS samples. **a**, **b** lncRNA (**a**) and mRNA (**b**) volcano plots of RA FLSs versus normal FLSs(*CONT*). Each *square* represents a different transcript. *Red squares* represent genes that passed the statistical and fold-change cutoffs. **c**, **d** lncRNA (**c**) and mRNA (**d**) heat maps showing distinguishable lncRNA expression profiles between patients with RA and patients with trauma. Each *column* indicates a different sample. Each *row* indicates one mRNA or lncRNA. Relatively high expression is indicated by *red shading* and relatively low expression is indicated by *green shading*

To visualize the differentially expressed lncRNAs and mRNAs, volcano plot analysis was conducted to further explore the difference between the RA FLSs and normal FLSs (Fig. 2a and b). The data showed that 135 (0.44 %) of the lncRNAs and 103 (0.39 %) of the mRNAs were significantly differentially expressed. The results of hierarchical cluster analyses showed distinguishable lncRNA and mRNA expression profiles between the RA FLSs and normal FLSs (Fig. 2c and d).

### Pathway and GO analyses

Pathway and GO analyses were applied to explore the potential roles that the differentially expressed mRNAs might play in RA. Pathway analysis showed that the differentially expressed mRNAs in RA FLSs were related with mTOR, HIF-1, Ras, proteoglycans, and apoptosis signaling pathways, among others (Additional file 4: Figure S1). These mRNAs were further characterized as being mainly involved in regulating biological processes related to cell growth, vascular permeability, and cell activation (Additional files 5 and 6: Figures S2 and S3).

### Classification of lncRNAs

**Fig. 3:**
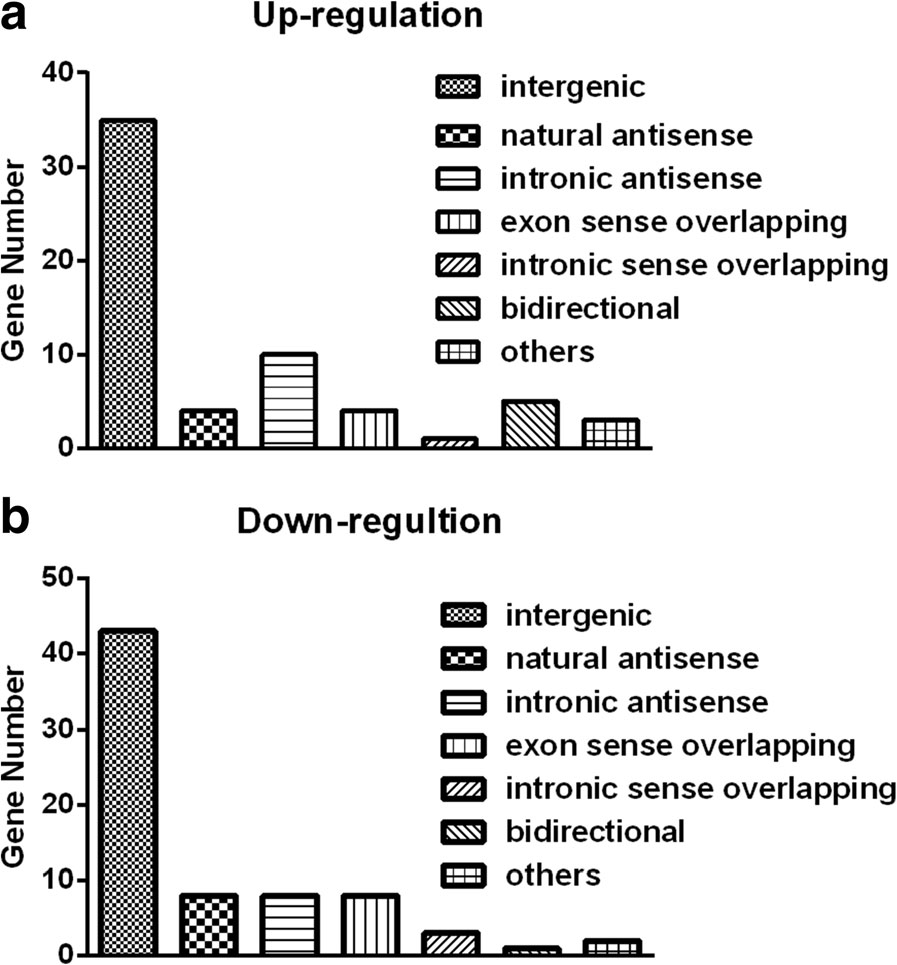
Classification analyses of up-regulated lncRNAs **a** (*Up-regulati*on) and down-regulated lncRNAs **b** (*Down-regulatio*n) in rheumatoid arthritis fibroblast-like synoviocytes, as detected by microarray analysis

Of the 62 up-regulated lncRNAs identified, we observed 4 (6.5 %) natural antisense, 10 (16.1 %) intronic antisense, 4 (6.5 %) exon sense overlapping, 1 (1.6 %) intron sense overlapping, 5 (8.1 %) bidirectional, 35 (56.5 %) intergenic, and 3 (4.8 %) other lncRNAs (Fig. 3a). Of the 73 down-regulated lncRNAs identified, we observed 8 (11.0 %) natural antisense, 8 (11.0 %) intronic antisense, 8 (11.0 %) exon sense overlapping, 3 (4.1 %) intron sense overlapping, 1 (1.4 %) bidirectional, 43 (58.9 %) intergenic, and 2 (2.7 %) other lncRNAs (Fig. 3b).

### qPCR validation

**Fig. 4:**
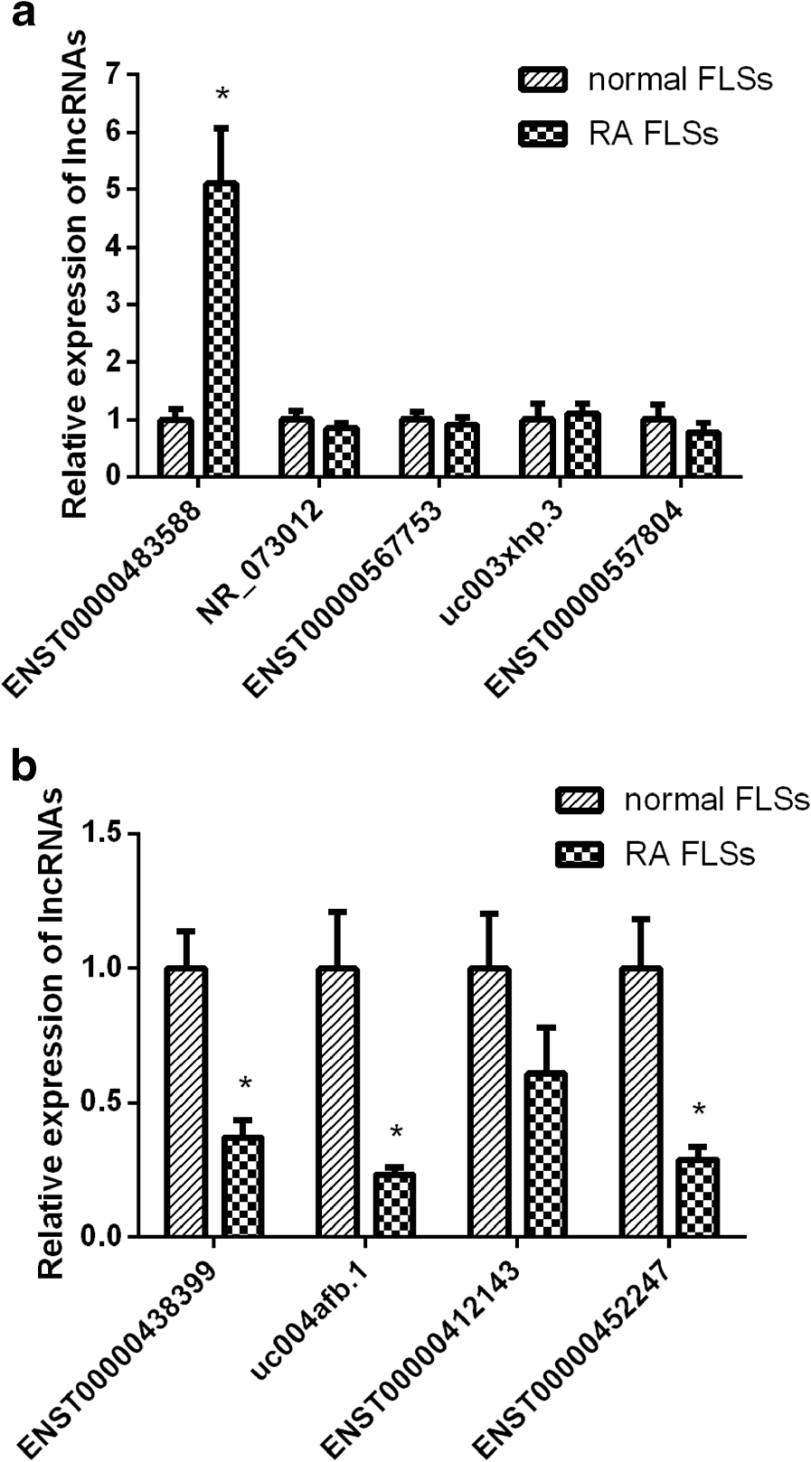
Relative expression of lncRNAs using qPCR. **a** Expression of ENST00000483588, NR_073012, ENST00000567753, uc003xhp.3, and ENST00000557804 in rheumatoid arthritis (*RA*) fibroblast-like synoviocyte (*FLS*) samples (n = 10) and normal FLS samples (n = 10). **b** Expression of ENST00000438399, uc004afb.1, ENST00000412143, and ENST00000452247 in RA FLS samples (n = 10) and normal FLS samples (n = 10). Values are the mean ± SEM; *P < 0.01 for RA versus normal FLS samples

Considering the observed fold changes, the calculated *P* values, and the primer specificities, we selected the following nine lncRNAs for further validation of expression levels by qPCR: ENST00000483588, NR_073012, ENST00000567753, uc003xhp.3, ENST00000557804, ENST00000438399, uc004afb.1, ENST00000412143, and ENST00000452247 (Table 1). The qPCR results confirmed that ENST00000483588 was overexpressed in the RA FLSs, whereas the expression levels of ENST00000438399, uc004afb.1, and ENST00000452247 were decreased in the RA FLSs (Fig. 4). These four lncRNAs showed similar trends in qPCR, as determined by microarray. Nevertheless, some lncRNAs showed no significant difference between RA FLSs and normal FLSs in qPCR in contrast to the results determined in the microarray.

**Table 1:** Nine differentially expressed lncRNAs in rheumatoid arthritis fibroblast-like synoviocytes (FLSs) versus normal FLSs determined by microarray

Sequence name	Gene symbol	*P* value	Fold change	Regulation
ENST00000483588	*C17orf76-AS1*	<0.01	8.91	up
NR_073012	*PRSS21*	<0.01	3.48	up
ENST00000567753	*RP11-524C21.2*	<0.01	3.38	up
uc003xhp.3	*BC015784*	<0.01	3.21	up
ENST00000557804	*AC068831.10*	<0.01	2.97	up
ENST00000438399	*RP11-534G20.3*	<0.01	16.41	down
uc004afb.1	*AK096159*	<0.01	7.30	down
ENST00000412143	*PSORS1C3*	<0.01	5.01	down
ENST00000452247	*RP11-573I11.2*	<0.01	4.22	down

### Correlation between the aberrantly expressed lncRNAs and clinical characteristics of patients with RA

**Fig. 5:**
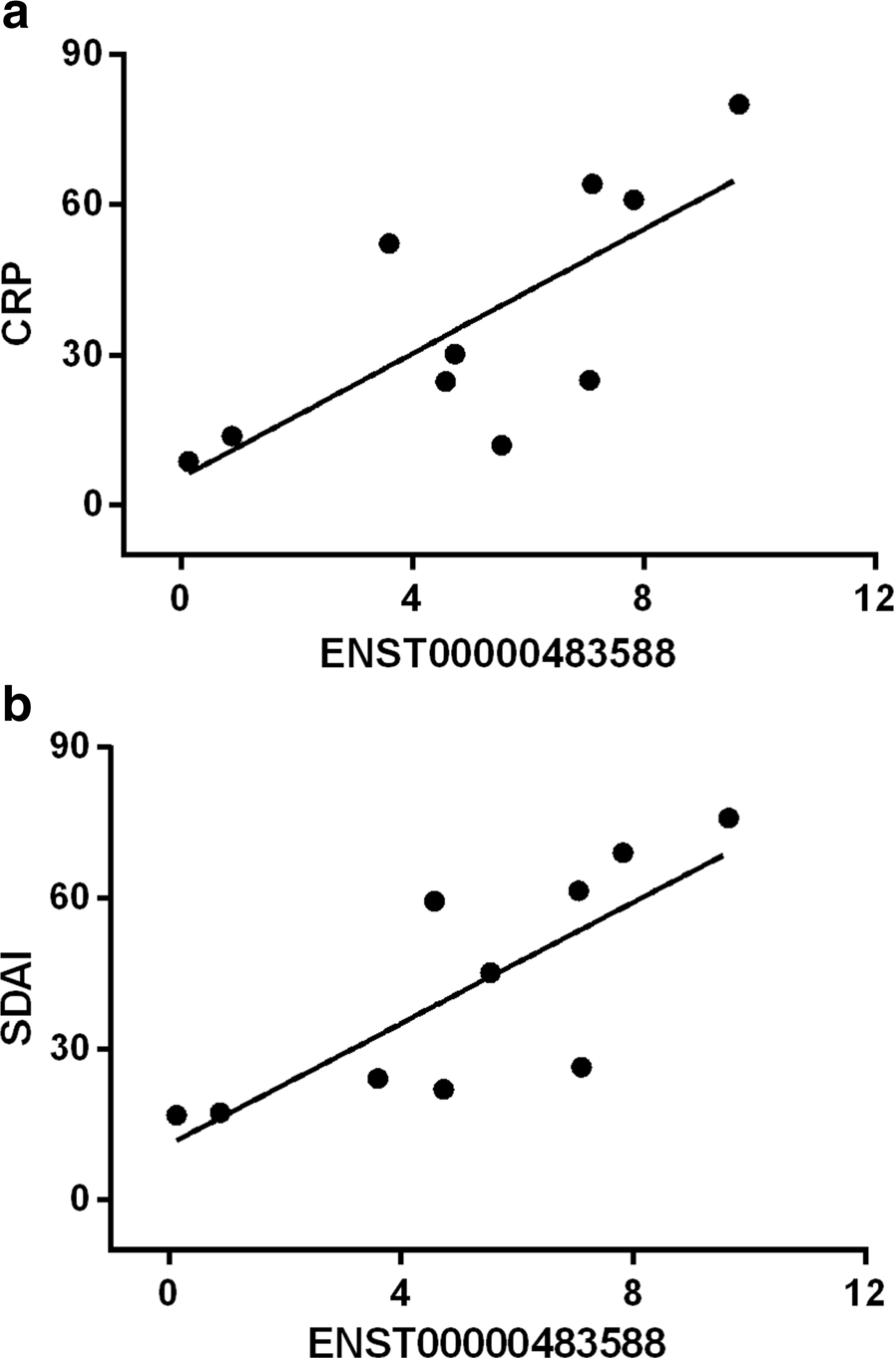
Correlation between lncRNAs and the clinical characteristics of patients with rheumatoid arthritis. **a** Correlation between ENST00000483588 expression and C-reactive protein (*CRP*). **b** Correlation between ENST00000483588 expression and the Simplified Disease Activity Index (*SDAI*) score

We further analyzed the correlation between the expression levels of the lncRNAs ENST00000483588, ENST00000438399, uc004afb.1, and ENST00000452247 and clinical characteristics (erythrocyte sedimentation rate (ESR), C-reactive protein (CRP) expression, Simplified Disease Activity Index (SDAI) score, and age). The expression level of ENST00000483588 was positively correlated with the CRP level (*r* = 0.74, *P* < 0.05) and the SDAI score (*r* = 0.79, *P* < 0.01) (Fig. 5). However, none of these lncRNAs were significantly correlated with ESR or age.

### Diagnostic value of four selected lncRNAs

**Fig. 6:**
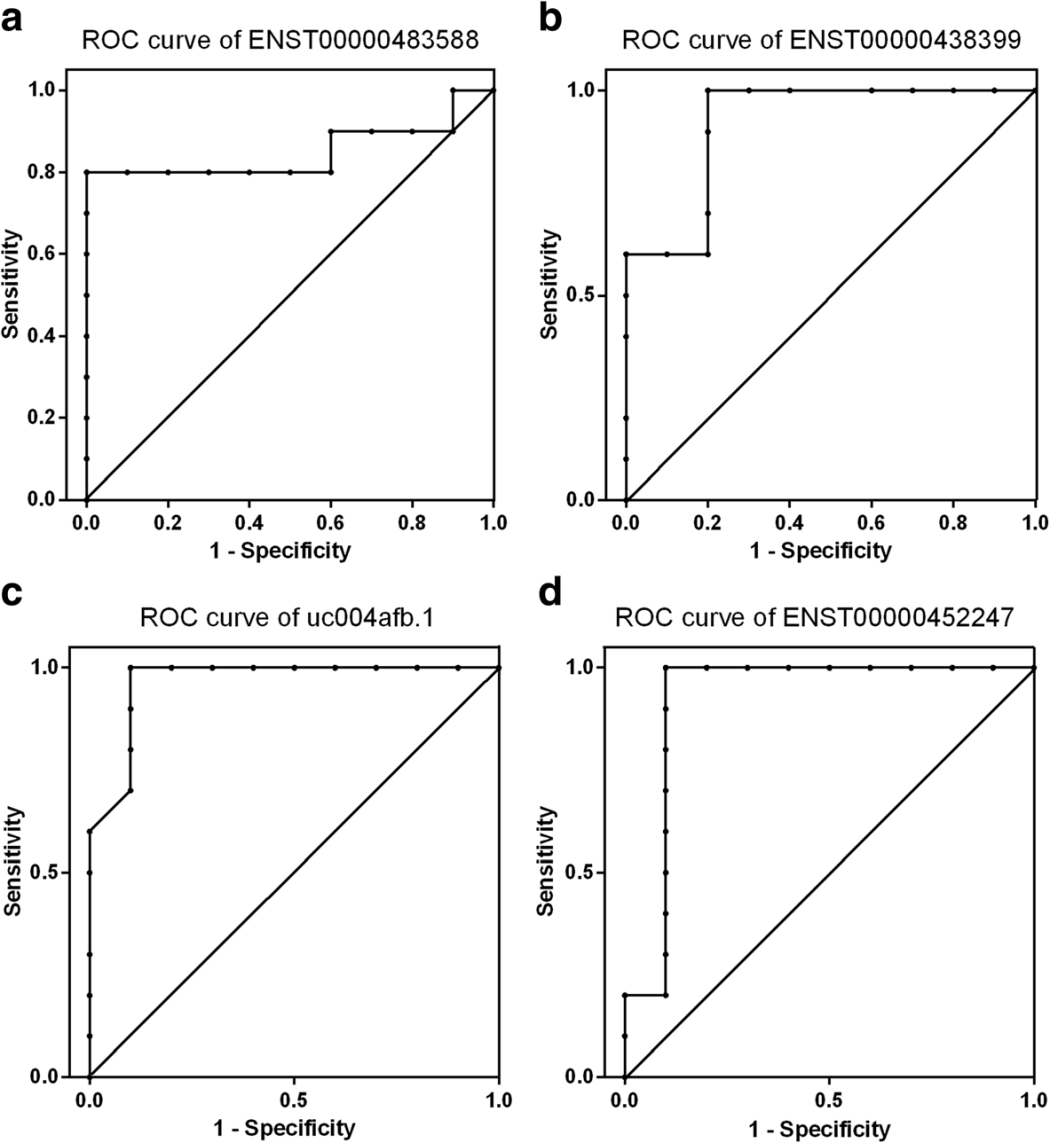
Receiver operating characteristic (*ROC*) curves. **a**–**d** ROC curves for patients rheumatoid arthritis (RA) based on the expression of ENST00000483588 (**a**), ENST00000438399 (**b**), uc004afb.1 (**c**), and ENST00000452247 (**d**) in RA fibroblast-like synoviocytes (FLSs) and normal FLSs

The potential diagnostic value of four lncRNAs for RA was evaluated using patients with trauma patients as control subjects. The area under the ROC curve for the ENST00000483588, ENST00000438399, uc004afb.1, and ENST00000452247 lncRNAs was 0.85, 0.92, 0.97, and 0.92, respectively, indicating potential diagnostic value for RA (Fig. 6).

### LncRNA and mRNA co-expression network

An lncRNA and mRNA co-expression network was constructed based on the correlation analysis between the differentially expressed lncRNAs and mRNAs. We further focused on co-expression networks centering on ENST00000483588, ENST00000438399, uc004afb.1, and ENST00000452247. The co-expression networks showed that ENST00000483588 expression was positively correlated with *ATAD3A* and *NDUFA4L2* mRNA expression levels (Additional file 7: Figure S4), ENST00000438399 expression was positively correlated with *PTPRQ* mRNA expression and negatively correlated with *PTHLH* mRNA expression (Additional file 8: Figure S5), uc004afb.1 expression was positively correlated with *TNFRSF11B* mRNA expression (Additional file 9: Figure S6), and ENST00000452247 expression was positively correlated with *ZNF154* mRNA expression and negatively correlated with *WISP3* mRNA expression (Additional file 10: Figure S7).

## Discussion

To date, expression profile studies of cells and tissues have mainly focused on mRNAs and microRNAs. Recent advances in the depth and quality of transcriptome sequencing have revealed an increasing number of distinguishably expressed lncRNAs in various diseases. Although several findings have implicated lncRNAs in the development and progression of various diseases, research on lncRNAs related to rheumatic diseases is limited. Liu et al. [15] demonstrated that lncRNA-CIR expression in chondrocytes promoted extracellular matrix degradation by affecting the expression of collagen, aggrecan, and matrix-degrading enzymes, and plays an important role in the pathogenesis of osteoarthritis. Furthermore, studies of lncRNAs in T cells [16] and monocytes [17, 18] from patients with RA have been conducted. The aim of our study was to explore lncRNA expression in RA FLSs to provide new insight into the pathogenesis of RA. A schematic diagram of our overall study design and main findings is provided in Additional file 11: Figure S8.

We analyzed three RA FLS samples and three normal FLS samples using lncRNA and mRNA microarrays. Based on the microarray data, we found 135 lncRNAs and 103 mRNAs that were differentially expressed. Most of these lncRNAs have not been functionally characterized, whereas most of the identified mRNAs are well-known. Therefore, bioinformatics analysis of the aberrantly expressed mRNAs was conducted to help better understand the potential role of FLSs in the pathological process of RA and speculate on the putative function of the differentially expressed lncRNAs, since previous reports have showed that lncRNAs participate in a wide variety of pathological processes by regulating gene expression at the levels of chromatin remodeling, transcriptional control, and post-transcriptional processing. GO and pathway analyses showed that the differentially expressed mRNAs mainly related to regulation of growth, vascular permeability, and cell activation processes that are clearly associated with RA pathogenesis [19–21].

We used qPCR to validate the lncRNA microarray results. Based on the qPCR results, ENST00000483588, ENST00000438399, uc004afb.1 and ENST00000452247 were differentially expressed, which was in agreement with the microarray results. Nevertheless, other lncRNAs did not differ significantly in RA FLSs and normal FLSs on qPCR, in contrast to the results from the microarray. The different trends are likely due to the fact that the expanded test sample size for the qPCR might have excluded some of the false positive results obtained in the microarray.

ENST00000483588 is a 689-bp intronic antisense lncRNA transcript from the *C17orf76-AS1* gene. As shown in Table 1 and Fig. 4, ENST00000483588 was significantly up-regulated in RA FLSs compared with normal FLSs. Nakaya et al. [22] showed that intronic antisense lncRNAs are enriched in the introns of genes related to regulation of transcription, and potentially function as regulators of alternative splicing. However, in the present study, the expression of *FAM211A*, the coding gene corresponding to the lncRNA ENST00000483588, was not significantly different between the RA FLSs and normal FLSs. ENST00000483588 has an antisense orientation relative to the coding gene, and the transcribed regions partially overlap without exons. The co-expression network of ENST00000483588 showed that ENST00000483588 expression was positively correlated with *ATAD3A* and *NDUFA4L2* mRNA expression. *ATAD3A* encodes a mitochondrial membrane protein that helps stabilize large mitochondrial DNA-protein complexes, known as nucleoids [23]. *NDUFA4L2* plays a role in the biological oxidation of mitochondria. Thus, the role of ENST00000483588 in mitochondrial function merits further study.

Interestingly, strong associations between ENST00000483588 expression and the CRP level and SDAI score in patients with RA were found. CRP is an important clinical parameter that is commonly used as a marker of disease activity in RA [11, 24, 25]. Recent studies have shown that CRP is not only a product of the inflammatory response but also plays a proinflammatory role in RA. CRP can activate complements and induce osteoclast differentiation [26, 27]. The CRP level can also provide an indication of treatment efficacy, with decreased CRP reflecting effective treatment against arthritis, and sustained high CRP reflecting a poor treatment response [28, 29]. The SDAI is a valid and sensitive composite index to assess disease activity and define cutoff values representing remission in RA, as recommended by the ACR and EULAR [30]. Considering the high diagnostic value of ENST00000483588, we speculate that this lncRNA may be involved in the pathological process of RA. Nevertheless, further studies are needed to clarify the underlying mechanisms.

In contrast to ENST00000483588, the expression level of ENST00000438399 in RA FLSs was markedly lower than that in normal FLSs. ENST00000438399 is a 3307-bp intergenic lncRNA transcript from the *RP11-534G20.3* gene located on chromosome 10: 29698475–29713107. The co-expression network of ENST00000438399 showed that ENST00000438399 expression was positively correlated with *PTPRQ* mRNA and negatively correlated with *PTHLH* mRNA. *PTPRQ* can inhibit cell proliferation, and induce apoptosis [31]. *PTHLH* can promote cell proliferation and exert a protective effect against apoptosis; moreover, its expression correlates with the severity of carcinoma [32]. In this study, the microarray analysis showed that the expression level of *PTPRQ* was down-regulated, whereas the expression level of *PTHLH* was up-regulated in RA FLSs. Previous reports have demonstrated that the human genome encodes at least 3289 long intergenic noncoding RNA, which are evolutionarily conserved and may be involved in diverse biological processes, including cell-cycle regulation, immune surveillance, and embryonic stem cell pluripotency [33, 34]. Whether ENST00000438399 interacts with *PTPRQ* or *PTHLH* needs to be verified, as does its role in regulating the growth of RA FLSs.

Moreover, the expression levels of uc004afb.1 and ENST00000452247 also decreased in RA FLSs. uc004afb.1 is a 2045-bp intergenic lncRNA transcript expressed from the *AK096159* gene located on chromosome 9: 68,743,530-68,769,869. The co-expression network of uc004afb.1 showed that uc004afb.1 was positively correlated with *TNFRSF11B* mRNA expression. *TNFRSF11B*, which encodes osteoprotegerin, functions as a negative regulator of bone resorption [35]. It was down-regulated in the RA FLSs in the microarray results. ENST00000452247 is an 869-bp natural antisense lncRNA transcript expressed from the *RP11-573I11.2* gene. The co-expression network of ENST00000452247 showed that ENST00000452247 expression was positively correlated with *ZNF154* mRNA expression and negatively correlated with *WISP3* mRNA expression. *ZNF154* encodes a protein that belongs to the Krüppel family of zinc finger transcriptional regulators, the members of which are thought to function in normal and abnormal cell growth and differentiation. Hypermethylation and low expression levels of *ZNF154* have been considered promising biological markers for tumor identification and cancer recurrence surveillance [36].

Abnormal expression of *WISP3* has been detected in a variety of tumors. Silencing *WISP3* expression suppresses cell proliferation and induces apoptosis in bladder cancer cells [37], and also suppresses cell proliferation and migration and Wnt signaling, and the expression of adhesion molecules in gastric cancer cells [38]. Moreover, mutations of this gene are associated with progressive pseudo-rheumatoid dysplasia [39, 40]. Hypomethylation and relatively high expression of *WISP3* have been demonstrated in a previous study [41]. In this study, the microarray analysis showed that the expression level of *WISP3* was up-regulated in RA FLSs. The pathological phenomena of bone destruction, angiogenesis, and abnormal proliferation of FLSs are characteristics of RA. Therefore, the results of the co-expression networks provide clues for further research into the molecular pathogenic mechanisms underlying RA. The lncRNAs uc004afb.1 and ENST00000452247 also had high diagnostic value, with areas under the ROC curve of 0.965 and 0.92, respectively. Nevertheless, the molecular mechanisms of uc004afb.1 and ENST00000452247 in RA require further study.

## Conclusions

In summary, our study provides comprehensive lncRNA and mRNA profiles for RA FLSs. The differential expression of ENST00000483588, ENST00000438399, uc004afb.1, and ENST00000452247 in RA FLSs suggests that these lncRNAs may participate in the pathogenesis of RA. The ROC curve analysis indicated that these lncRNAs may also have diagnostic value for RA. Expanding the RA FLS samples and adding plasma sample detection would help to clarify their suitability in clinical diagnosis. Although these results are preliminary at this stage, they are valuable in expanding knowledge on the role of lncRNAs in rheumatic diseases, and providing targets for further research, specifically on their link to the pathogenesis of RA.

## Additional files

Additional file 1: Table S1. Clinical characteristic of patients with rheumatoid arthritis (RA) and trauma. (DOC 45 kb)
Additional file 2: Table S2. Differentially expressed lncRNAs in RA FLSs versus normal FLSs. (DOC 219 kb)
Additional file 3: Table S3. Differentially expressed mRNAs in RA FLSs versus normal FLSs. (DOC 174 kb)
Additional file 4: Figure S1. Pathway analysis for differentially expressed (*DE*) mRNAs. *a* Pathway analysis for up-regulated mRNAs. *b* Pathway analysis for down-regulated mRNAs. (TIF 259 kb)
Additional file 5: Figure S2. Gene ontology (GO) analysis for up-regulated mRNAs. **a** Biological process (*BP*) analysis for up-regulated mRNAs. **b** Cellular component (*CC*) analysis for up-regulated mRNAs. **c** Molecular function (*MF*) analysis for up-regulated mRNAs. (TIF 432 kb)
Additional file 6: Figure S3. Gene ontology (GO) analysis for down-regulated mRNAs. **a** Biological process (*BP*) analysis for down-regulated mRNAs. **b** Cellular component (*CC*) analysis for down-regulated mRNAs. **c** Molecular function (*MF*) analysis for down-regulated mRNAs. (TIF 468 kb)
Additional file 7: Figure S4. Co-expression network of the differentially expressed lncRNAs and mRNAs. ENST00000483588 was connected to 12 lncRNAs and 9 mRNAs. *Blue nodes* represent lncRNAs and *yellow nodes* represent protein-coding genes. A *red line* represents a positive correlation, and a *green line* represents negative correlation. (TIF 863 kb)
Additional file 8: Figure S5. Co-expression network of the differentially expressed lncRNAs and mRNAs. ENST00000438399 was connected to 11 lncRNAs and 9 mRNAs. *Blue nodes* represent lncRNAs and *yellow nodes* represent protein-coding genes. A *red line* represents positive correlation, and a *green line* represents negative correlation. (TIF 756 kb)
Additional file 9: Figure S6. Co-expression network of the differentially expressed lncRNAs and mRNAs. uc004afb.1 was connected to 17 lncRNAs and 8 mRNAs. *Blue nodes* represent lncRNAs and *yellow nodes* represent protein-coding genes. A *red line* represents positive correlation, and a *green line* represents negative correlation. (TIF 889 kb)
Additional file 10: Figure S7. Co-expression network of the differentially expressed lncRNAs and mRNAs. ENST00000452247 was connected to 18 lncRNAs and 12 mRNAs. *Blue nodes* represent lncRNAs and *yellow nodes* represent protein-coding genes. A *red line* represents positive correlation, and a *green line* represents negative correlation. (TIF 1529 kb)
Additional file 11: Figure S8. Schematic diagram of the study design and summary of main findings. (TIF 814 kb)

## Abbreviations

ACR/EULAR: American College of Rheumatology/European league Against Rheumatism
CRP: C-reactive protein
DMEM: Dulbecco’s modified Eagle’s medium
bp: base pair(s)
ESR: erythrocyte sedimentation rate
FBS: fetal bovine serum
FITC: fluorescein isothiocyanate
FLSs: fibroblast-like synoviocytes
GO: Gene Ontology
lncRNA: long noncoding RNA
ncRNA: non-coding RNA
PBS: phosphate-buffered saline
qPCR: quantitative polymerase chain reaction
RA: rheumatoid arthritis
ROC: receiver operating characteristic
SDAI: Simplified Disease Activity Index
