# Supplementary material for: Long noncoding RNA expression profile in fibroblast-like synoviocytes from patients with rheumatoid arthritis
Source: Arthritis Res Ther. 2016 Oct 6;18:227. doi: 10.1186/s13075-016-1129-4 (PMC5053204; doi:10.1186/s13075-016-1129-4)
Supplement: Additional file 1: Table S1. — Clinical characteristic of patients with rheumatoid arthritis (RA) and trauma. (DOC 45 kb) [file 13075_2016_1129_MOESM1_ESM.doc]

**Additional file 1: Table S1.** Clinical characteristic of rheumatoid arthritis(RA) and trauma patients.

| Clinical data | RA patients | Trauma patients |
| --- | --- | --- |
| male | 2 | 3 |
| female | 8 | 7 |
| Age (years) | 52.4*±*8.2 | 47.1*±*7.9 |
| Disease duration (years) | 11.2±6.5 | 0.51*±*0.60 |
| Tender joints^a^ | 11.7±10.5 | 1 |
| Swollen joints^a^ | 11.4±10.2 | 1 |
| Patients assessment^b^ | 7.9±1.3 | / |
| Physician assessment^b^ | 7.1±0.88 | / |
| CRP (mg/dl) | 37.2±25.1 | 2.8±5.5 |
| ESR (mm/h) | 37.9±22.8 | / |
| SDAI | 41.8±23.0 | / |
| Treatments  DMARD (n)  NSAID (n)  Corticosteroid (n) | 9  5  7 | none  none  none |

^a^ Twenty-eight joints were assessed for tenderness, and twenty-eight were assessed for swelling. ^b^ A 0–10 visual-analogue scale was used in which higher values indicated more severe abnormalities. *DMARD* disease-modifying anti-rheumatic drug, *NSAID* non-steroidal anti-inflammatory drug, *n* numble of patients
