# Supplementary material for: Long noncoding RNA expression profile in fibroblast-like synoviocytes from patients with rheumatoid arthritis
Source: Arthritis Res Ther. 2016 Oct 6;18:227. doi: 10.1186/s13075-016-1129-4 (PMC5053204; doi:10.1186/s13075-016-1129-4)
Supplement: Additional file 2: Table S2. — Differentially expressed lncRNAs in RA FLSs versus normal FLSs. (DOC 219 kb) [file 13075_2016_1129_MOESM2_ESM.doc]

**Additional file 2：Table S2.** Differentially expressed lncRNAs in RA FLSs versus normal FLSs.

| seqname | GeneSymbol | *P*-value | Fold Change | Regulation |
| --- | --- | --- | --- | --- |
| ENST00000421322 | *XIST* | 0.005 | 657.36 | up |
| uc004ebm.1 | *XIST* | 0.004 | 647.91 | up |
| ENST00000434839 | *XIST* | 0.006 | 639.51 | up |
| ENST00000417942 | *XIST* | 0.001 | 185.19 | up |
| ENST00000416330 | *XIST* | 0.007 | 36.98 | up |
| ENST00000472367 | *C17orf76-AS1* | 0.003 | 25.05 | up |
| ENST00000478103 | *C17orf76-AS1* | 0.000 | 17.83 | up |
| NR_027177 | *C17orf76-AS1* | 0.003 | 10.72 | up |
| ENST00000483588 | *C17orf76-AS1* | 0.002 | 8.91 | up |
| ENST00000437987 | *RP4-715N11.2* | 0.019 | 8.17 | up |
| ENST00000425279 | *RP4-715N11.2* | 0.040 | 5.71 | up |
| ENST00000490320 | *RP11-431I8.1* | 0.021 | 4.53 | up |
| ENST00000542000 | *RP11-143E21.7* | 0.046 | 4.41 | up |
| ENST00000527726 | *RP11-483L5.1* | 0.044 | 3.99 | up |
| ENST00000562490 | *RP11-102F4.3* | 0.033 | 3.87 | up |
| uc003ndl.3 | *LINC00340* | 0.008 | 3.87 | up |
| uc002utn.1 | *BC062769* | 0.012 | 3.84 | up |
| NR_073012 | *PRSS21* | 0.002 | 3.48 | up |
| HMlincRNA1594+ | *HMlincRNA1594* | 0.038 | 3.48 | up |
| ENST00000567753 | *RP11-524C21.2* | 0.034 | 3.39 | up |
| uc001jbz.3 | *LOC100133308* | 0.045 | 3.26 | up |
| uc021uup.1 | *LGALS17A* | 0.049 | 3.23 | up |
| uc003xhp.3 | *BC015784* | 0.013 | 3.22 | up |
| uc001uih.2 | *FLJ31485* | 0.037 | 3.05 | up |
| ENST00000557804 | *AC068831.10* | 0.001 | 2.97 | up |
| ENST00000503989 | *CTD-2044J15.1* | 0.038 | 2.96 | up |
| NR_026706 | *LOC286094* | 0.026 | 2.95 | up |
| ENST00000334697 | *LINC00470* | 0.008 | 2.94 | up |
| ENST00000432673 | *RP11-390P2.4* | 0.025 | 2.89 | up |
| ENST00000447835 | *AC012462.2* | 0.020 | 2.89 | up |
| ENST00000370257 | *RP11-261N11.8* | 0.017 | 2.78 | up |
| ENST00000570105 | *RP11-107D24.2* | 0.015 | 2.74 | up |
| ENST00000562248 | *LINC00304* | 0.012 | 2.74 | up |
| ENST00000434730 | *KB-1183D5.14* | 0.044 | 2.74 | up |
| ENST00000455931 | *RP11-348F1.2* | 0.047 | 2.73 | up |
| ENST00000427610 | *RP11-399K21.10* | 0.042 | 2.71 | up |
| TCONS_00027643 | *XLOC_013182* | 0.020 | 2.71 | up |
| uc003ndk.1 | *LINC00340* | 0.023 | 2.65 | up |
| ENST00000541103 | *SCAND2* | 0.005 | 2.63 | up |
| ENST00000419064 | *RP11-13P5.2* | 0.033 | 2.57 | up |
| uc001iuq.1 | *LOC387647* | 0.039 | 2.50 | up |
| ENST00000564127 | *RP11-480I12.10* | 0.019 | 2.32 | up |
| ENST00000446167 | *RP11-7O11.3* | 0.005 | 2.31 | up |
| NR_034095 | *LOC400456* | 0.030 | 2.29 | up |
| ENST00000457449 | *RP11-329B9.1* | 0.011 | 2.26 | up |
| ENST00000417281 | *CTC-338M12.4* | 0.046 | 2.24 | up |
| ENST00000443234 | *RP11-14I4.3* | 0.034 | 2.22 | up |
| ENST00000578391 | *RP11-286N3.2* | 0.017 | 2.22 | up |
| TCONS_00013408 | *XLOC_006039* | 0.020 | 2.21 | up |
| ENST00000567242 | *AC141586.5* | 0.029 | 2.20 | up |
| ENST00000540761 | *RP11-335O4.1* | 0.042 | 2.20 | up |
| ENST00000580113 | *KRT16P3* | 0.022 | 2.19 | up |
| TCONS_00012343 | *XLOC_005895* | 0.027 | 2.16 | up |
| ENST00000510311 | *AC005592.2* | 0.033 | 2.15 | up |
| uc004ebo.2 | *JPX* | 0.014 | 2.15 | up |
| NR_027409 | *GOLGA8A* | 0.023 | 2.11 | up |
| ENST00000558427 | *RP11-66B24.2* | 0.013 | 2.09 | up |
| ENST00000437201 | *SBDSP1* | 0.001 | 2.07 | up |
| NR_033917 | *LOC728228* | 0.039 | 2.05 | up |
| uc022agm.1 | *BC063788* | 0.049 | 2.04 | up |
| ENST00000429872 | *AC147651.4* | 0.009 | 2.04 | up |
| ENST00000415215 | *JPX* | 0.022 | 2.04 | up |
| NR_001545 | *TTTY15* | 0.000 | 392.88 | down |
| ENST00000454875 | *TTTY14* | 0.000 | 79.23 | down |
| NR_001544 | *NCRNA00185* | 0.000 | 42.29 | down |
| NR_047624 | *UTY* | 0.000 | 37.28 | down |
| ENST00000417071 | *TTTY15* | 0.003 | 22.63 | down |
| NR_047626 | *UTY* | 0.004 | 20.25 | down |
| uc022coe.1 | *BC062752* | 0.001 | 17.91 | down |
| ENST00000438399 | *RP11-534G20.3* | 0.028 | 16.41 | down |
| NR_047616 | *UTY* | 0.027 | 15.71 | down |
| ENST00000533551 | *PRKY* | 0.000 | 13.08 | down |
| TCONS_00017629 | *XLOC_008277* | 0.012 | 8.52 | down |
| ENST00000452584 | *TTTY14* | 0.013 | 7.95 | down |
| uc004afb.1 | *AK096159* | 0.008 | 7.30 | down |
| NR_047598 | *UTY* | 0.008 | 7.25 | down |
| TCONS_00017630 | *XLOC_008282* | 0.005 | 6.97 | down |
| ENST00000449092 | *BX248398.1* | 0.004 | 5.53 | down |
| ENST00000417305 | *ZFY-AS1* | 0.008 | 5.45 | down |
| ENST00000412143 | *PSORS1C3* | 0.018 | 5.01 | down |
| ENST00000449362 | *AC114814.3* | 0.014 | 4.90 | down |
| NR_038358 | *LINC00640* | 0.035 | 4.83 | down |
| ENST00000550268 | *RP1-78O14.1* | 0.045 | 4.43 | down |
| ENST00000505047 | *AC007379.5* | 0.000 | 4.23 | down |
| ENST00000452247 | *RP11-573I11.2* | 0.005 | 4.22 | down |
| NR_047613 | *UTY* | 0.000 | 4.00 | down |
| ENST00000450803 | *RP11-27I1.2* | 0.013 | 3.96 | down |
| ENST00000512486 | *CTD-2154I11.2* | 0.028 | 3.85 | down |
| ENST00000430052 | *RP11-277L2.2* | 0.041 | 3.66 | down |
| TCONS_00001281 | *XLOC_000567* | 0.029 | 3.46 | down |
| ENST00000435115 | *RP11-403I13.4* | 0.008 | 3.33 | down |
| ENST00000534891 | *RP11-712B9.2* | 0.034 | 3.30 | down |
| ENST00000503140 | *RP11-148L24.1* | 0.026 | 3.30 | down |
| uc004acr.1 | *AK094644* | 0.040 | 3.19 | down |
| ENST00000572124 | *RP11-504P24.6* | 0.031 | 3.16 | down |
| uc004acs.2 | *DQ587119* | 0.003 | 3.03 | down |
| ENST00000425982 | *AC005392.13* | 0.044 | 3.01 | down |
| ENST00000582940 | *RP11-160O5.1* | 0.010 | 2.99 | down |
| NR_038387 | *LOC730091* | 0.037 | 2.98 | down |
| ENST00000513892 | *RP11-468H14.2* | 0.016 | 2.97 | down |
| uc003zfx.3 | *AY343892* | 0.010 | 2.96 | down |
| uc001yvp.4 | *HERC2P2* | 0.036 | 2.92 | down |
| TCONS_00003974 | *XLOC_001788* | 0.031 | 2.78 | down |
| ENST00000416700 | *RP11-58A12.3* | 0.037 | 2.78 | down |
| TCONS_00021755 | *XLOC_010350* | 0.026 | 2.74 | down |
| ENST00000444499 | *AL592284.1* | 0.019 | 2.74 | down |
| ENST00000433106 | *AL078621.4* | 0.049 | 2.73 | down |
| ENST00000437785 | *AL592284.1* | 0.031 | 2.59 | down |
| uc003ipy.1 | *AK094909* | 0.029 | 2.58 | down |
| ENST00000452057 | *AC007038.7* | 0.016 | 2.55 | down |
| NR_036584 | *LOC100289650* | 0.029 | 2.54 | down |
| ENST00000425674 | *RP11-493K19.3* | 0.026 | 2.48 | down |
| ENST00000446595 | *AL078621.4* | 0.022 | 2.47 | down |
| uc002tjx.4 | *AY343891* | 0.047 | 2.43 | down |
| ENST00000431553 | *RP11-69E11.4* | 0.023 | 2.42 | down |
| ENST00000449800 | *RP11-508M1.3* | 0.050 | 2.38 | down |
| NR_045623 | *NABP1* | 0.024 | 2.32 | down |
| TCONS_00002079 | *XLOC_000266* | 0.026 | 2.31 | down |
| ENST00000421735 | *U73167.7* | 0.012 | 2.31 | down |
| ENST00000518932 | *RP11-152P17.2* | 0.001 | 2.31 | down |
| ENST00000419931 | *RP11-445H22.4* | 0.045 | 2.28 | down |
| TCONS_00012127 | *XLOC_005657* | 0.011 | 2.28 | down |
| ENST00000466668 | *GUSBP2* | 0.040 | 2.27 | down |
| TCONS_00006553 | *XLOC_003177* | 0.007 | 2.25 | down |
| ENST00000414404 | *AC078941.1* | 0.048 | 2.22 | down |
| ENST00000428309 | *AL592284.1* | 0.034 | 2.19 | down |
| ENST00000527459 | *CD99P1* | 0.043 | 2.19 | down |
| ENST00000557518 | *LINC00519* | 0.023 | 2.18 | down |
| ENST00000556503 | *RP11-298I3.4* | 0.006 | 2.18 | down |
| ENST00000569226 | *HERC2P9* | 0.003 | 2.16 | down |
| ENST00000456620 | *AL592284.1* | 0.016 | 2.13 | down |
| ENST00000509144 | *RP11-152P17.2* | 0.047 | 2.09 | down |
| TCONS_00001489 | *XLOC_000828* | 0.048 | 2.06 | down |
| ENST00000458394 | *RP11-27I1.2* | 0.010 | 2.06 | down |
| ENST00000523724 | *RP11-17A4.3* | 0.042 | 2.02 | down |
