# Supplementary material for: Long noncoding RNA expression profile in fibroblast-like synoviocytes from patients with rheumatoid arthritis
Source: Arthritis Res Ther. 2016 Oct 6;18:227. doi: 10.1186/s13075-016-1129-4 (PMC5053204; doi:10.1186/s13075-016-1129-4)
Supplement: Additional file 3: Table S3. — Differentially expressed mRNAs in RA FLSs versus normal FLSs. (DOC 174 kb) [file 13075_2016_1129_MOESM3_ESM.doc]

**Additional file 3: Table S3.** Differentially expressed mRNAs in RA FLSs versus normal FLSs.

| **seqname** | **GeneSymbol** | ***P*-value** | **Fold Change** | **Regulation** |
| --- | --- | --- | --- | --- |
| ENST00000539839 | *FZD10* | 0.024 | 13.61 | up |
| NM_013996 | *TAC1* | 0.007 | 11.30 | up |
| NM_013997 | *TAC1* | 0.010 | 9.23 | up |
| ENST00000383490 | *TMEM200C* | 0.007 | 8.93 | up |
| NM_020142 | *NDUFA4L2* | 0.012 | 7.00 | up |
| NM_198239 | *WISP3* | 0.016 | 5.18 | up |
| ENST00000480798 | *CST7* | 0.048 | 4.61 | up |
| NM_005950 | *MT1G* | 0.036 | 4.35 | up |
| NM_003182 | *TAC1* | 0.017 | 4.23 | up |
| NM_006399 | *BATF* | 0.042 | 4.02 | up |
| NM_171998 | *RAB39B* | 0.050 | 3.89 | up |
| NM_001126133 | *TNNT1* | 0.024 | 3.65 | up |
| NM_001130159 | *STOX1* | 0.029 | 3.61 | up |
| NM_000921 | *PDE3A* | 0.018 | 3.56 | up |
| ENST00000331555 | *C1orf186* | 0.033 | 3.51 | up |
| NM_001170535 | *ATAD3A* | 0.001 | 3.41 | up |
| NM_001033756 | *VEGFA* | 0.045 | 3.12 | up |
| NM_020182 | *PMEPA1* | 0.011 | 3.08 | up |
| NM_145117 | *NAV2* | 0.043 | 3.04 | up |
| ENST00000321660 | *GJD4* | 0.004 | 2.93 | up |
| NM_001025368 | *VEGFA* | 0.032 | 2.86 | up |
| NM_001025369 | *VEGFA* | 0.046 | 2.85 | up |
| NM_005213 | *CSTA* | 0.016 | 2.65 | up |
| NM_005952 | *MT1X* | 0.022 | 2.58 | up |
| NM_024603 | *BEND5* | 0.001 | 2.54 | up |
| NM_199170 | *PMEPA1* | 0.016 | 2.53 | up |
| NM_005608 | *PTPRCAP* | 0.047 | 2.46 | up |
| NM_199169 | *PMEPA1* | 0.035 | 2.38 | up |
| NM_000618 | *IGF1* | 0.015 | 2.38 | up |
| NM_198966 | *PTHLH* | 0.024 | 2.19 | up |
| NM_001184776 | *SEZ6L* | 0.011 | 2.19 | up |
| NM_153706 | *SETD9* | 0.034 | 2.17 | up |
| ENST00000381799 | *RHOH* | 0.004 | 2.11 | up |
| NM_012110 | *CHIC2* | 0.008 | 2.07 | up |
| NM_001312 | *CRIP2* | 0.002 | 2.05 | up |
| NM_001184747 | *PAFAH1B2* | 0.035 | 2.01 | up |
| NM_001008 | *RPS4Y1* | 0.005 | 2830.26 | down |
| ENST00000361365 | *EIF1AY* | 0.000 | 1347.90 | down |
| ENST00000407724 | *CYorf15A* | 0.000 | 259.66 | down |
| ENST00000382832 | *CYorf15B* | 0.000 | 158.20 | down |
| ENST00000297967 | *NLGN4Y* | 0.000 | 102.44 | down |
| NM_001039567 | *RPS4Y2* | 0.001 | 78.53 | down |
| ENST00000443026 | *ABCB5* | 0.033 | 10.37 | down |
| NM_004654 | *USP9Y* | 0.046 | 10.12 | down |
| NM_001184825 | *PSG1* | 0.027 | 9.90 | down |
| ENST00000306511 | *PSG8* | 0.027 | 7.84 | down |
| ENST00000244296 | *PSG1* | 0.018 | 7.70 | down |
| NM_033358 | *CASP8* | 0.019 | 7.64 | down |
| NM_001145026 | *PTPRQ* | 0.046 | 6.92 | down |
| NM_001955 | *EDN1* | 0.040 | 6.79 | down |
| NM_016179 | *TRPC4* | 0.006 | 5.56 | down |
| NM_005044 | *PRKX* | 0.000 | 5.22 | down |
| NM_003465 | *CHIT1* | 0.044 | 4.88 | down |
| NM_001135956 | *TRPC4* | 0.034 | 4.55 | down |
| NM_001135955 | *TRPC4* | 0.011 | 4.53 | down |
| NM_007125 | *UTY* | 0.029 | 4.14 | down |
| NM_033143 | *FGF5* | 0.037 | 3.81 | down |
| NM_000933 | *PLCB4* | 0.009 | 3.78 | down |
| NM_182797 | *PLCB4* | 0.017 | 3.66 | down |
| NM_005118 | *TNFSF15* | 0.015 | 3.55 | down |
| NM_004932 | *CDH6* | 0.025 | 3.54 | down |
| ENST00000343958 | *FGD6* | 0.042 | 3.38 | down |
| NM_001136000 | *ABL2* | 0.033 | 3.35 | down |
| ENST00000537559 | *VEPH1* | 0.023 | 3.28 | down |
| NM_001004019 | *FBLN2* | 0.006 | 3.14 | down |
| NM_001038603 | *MARVELD2* | 0.012 | 3.13 | down |
| NM_001145453 | *GFRA1* | 0.026 | 3.11 | down |
| NM_001099401 | *SGCE* | 0.037 | 3.04 | down |
| NM_002546 | *TNFRSF11B* | 0.006 | 3.04 | down |
| NM_001126057 | *DMKN* | 0.028 | 3.03 | down |
| NM_052946 | *NOSTRIN* | 0.043 | 3.01 | down |
| NM_001710 | *CFB* | 0.030 | 2.96 | down |
| NM_152584 | *HSFY1* | 0.007 | 2.96 | down |
| NM_001190349 | *DMKN* | 0.030 | 2.92 | down |
| NM_001171632 | *NOSTRIN* | 0.028 | 2.90 | down |
| NM_033317 | *DMKN* | 0.030 | 2.83 | down |
| ENST00000355285 | *APCDD1* | 0.021 | 2.82 | down |
| NM_005264 | *GFRA1* | 0.048 | 2.78 | down |
| NM_198682 | *GYPE* | 0.038 | 2.74 | down |
| NM_001033719 | *ZNF404* | 0.037 | 2.70 | down |
| NM_001085384 | *ZNF154* | 0.003 | 2.70 | down |
| NM_024686 | *TTLL7* | 0.048 | 2.67 | down |
| NM_172212 | *CSF1* | 0.002 | 2.66 | down |
| NM_001172646 | *PLCB4* | 0.021 | 2.61 | down |
| NM_199441 | *ZNF334* | 0.050 | 2.59 | down |
| NM_001126058 | *DMKN* | 0.025 | 2.57 | down |
| NM_001099400 | *SGCE* | 0.024 | 2.53 | down |
| NM_001135957 | *TRPC4* | 0.007 | 2.45 | down |
| NM_058229 | *FBXO32* | 0.022 | 2.44 | down |
| NM_020700 | *PPM1H* | 0.003 | 2.44 | down |
| NM_004102 | *FABP3* | 0.028 | 2.43 | down |
| NM_182659 | *UTY* | 0.025 | 2.31 | down |
| NM_002514 | *NOV* | 0.044 | 2.30 | down |
| NM_004437 | *EPB41* | 0.046 | 2.29 | down |
| NM_003919 | *SGCE* | 0.012 | 2.28 | down |
| NM_001135775 | *CACFD1* | 0.037 | 2.26 | down |
| NM_032892 | *FRMD5* | 0.010 | 2.23 | down |
| NM_001101676 | *SAMD12* | 0.049 | 2.22 | down |
| NM_153283 | *HYAL1* | 0.002 | 2.17 | down |
| NM_018295 | *TMEM140* | 0.032 | 2.15 | down |
| ENST00000343684 | *NKAPL* | 0.013 | 2.06 | down |
| NM_000023 | *SGCA* | 0.039 | 2.05 | down |
| NM_001144823 | *DENND4A* | 0.037 | 2.04 | down |
