# Supplementary figures and images for: Long noncoding RNA expression profile in fibroblast-like synoviocytes from patients with rheumatoid arthritis
Source: Arthritis Res Ther. 2016 Oct 6;18:227. doi: 10.1186/s13075-016-1129-4 (PMC5053204; doi:10.1186/s13075-016-1129-4)

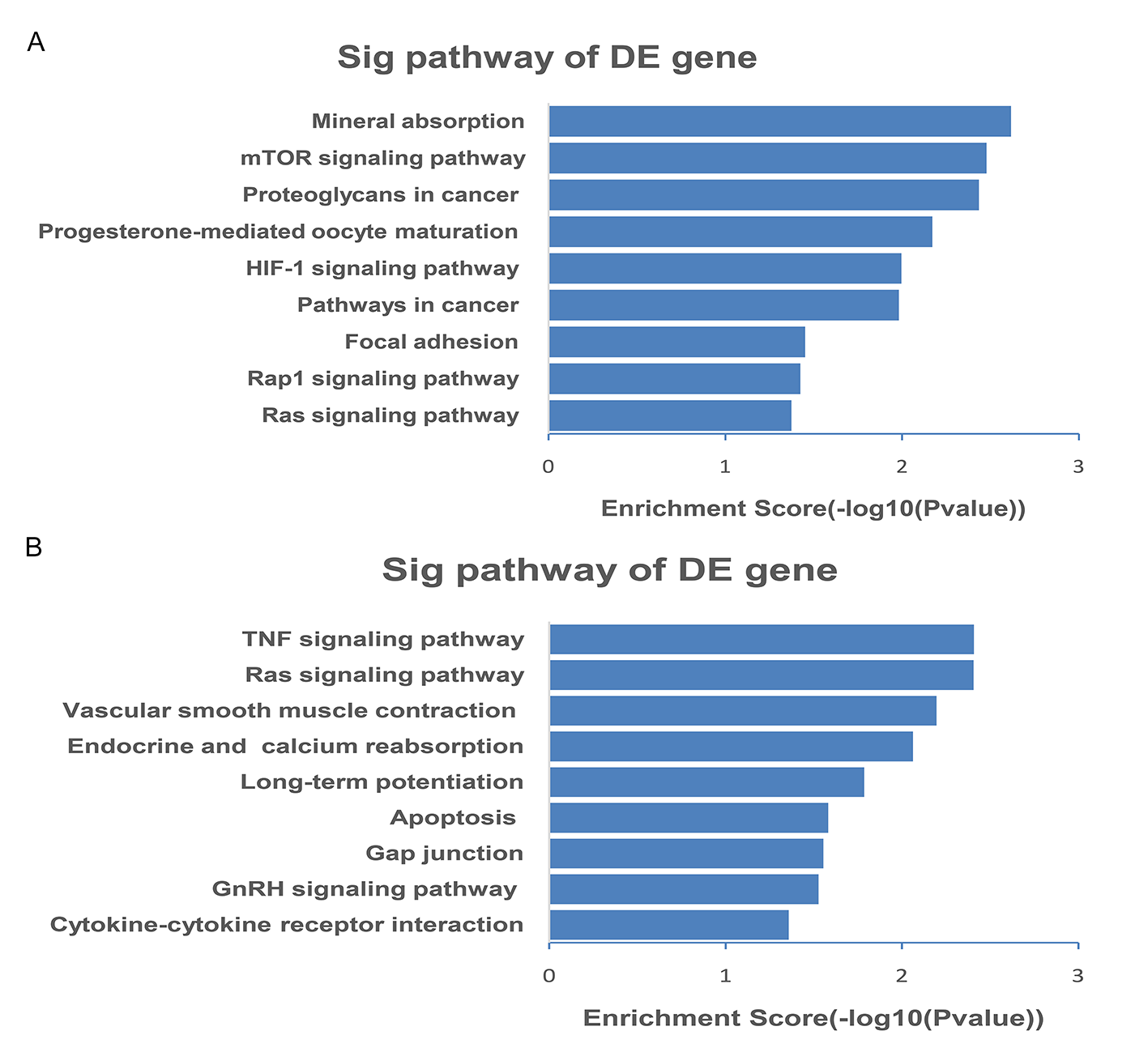

Supplement: Additional file 4: Figure S1. — Pathway analysis for differentially expressed (DE) mRNAs. a Pathway analysis for up-regulated mRNAs. b Pathway analysis for down-regulated mRNAs. (TIF 259 kb) [file 13075_2016_1129_MOESM4_ESM.tif]

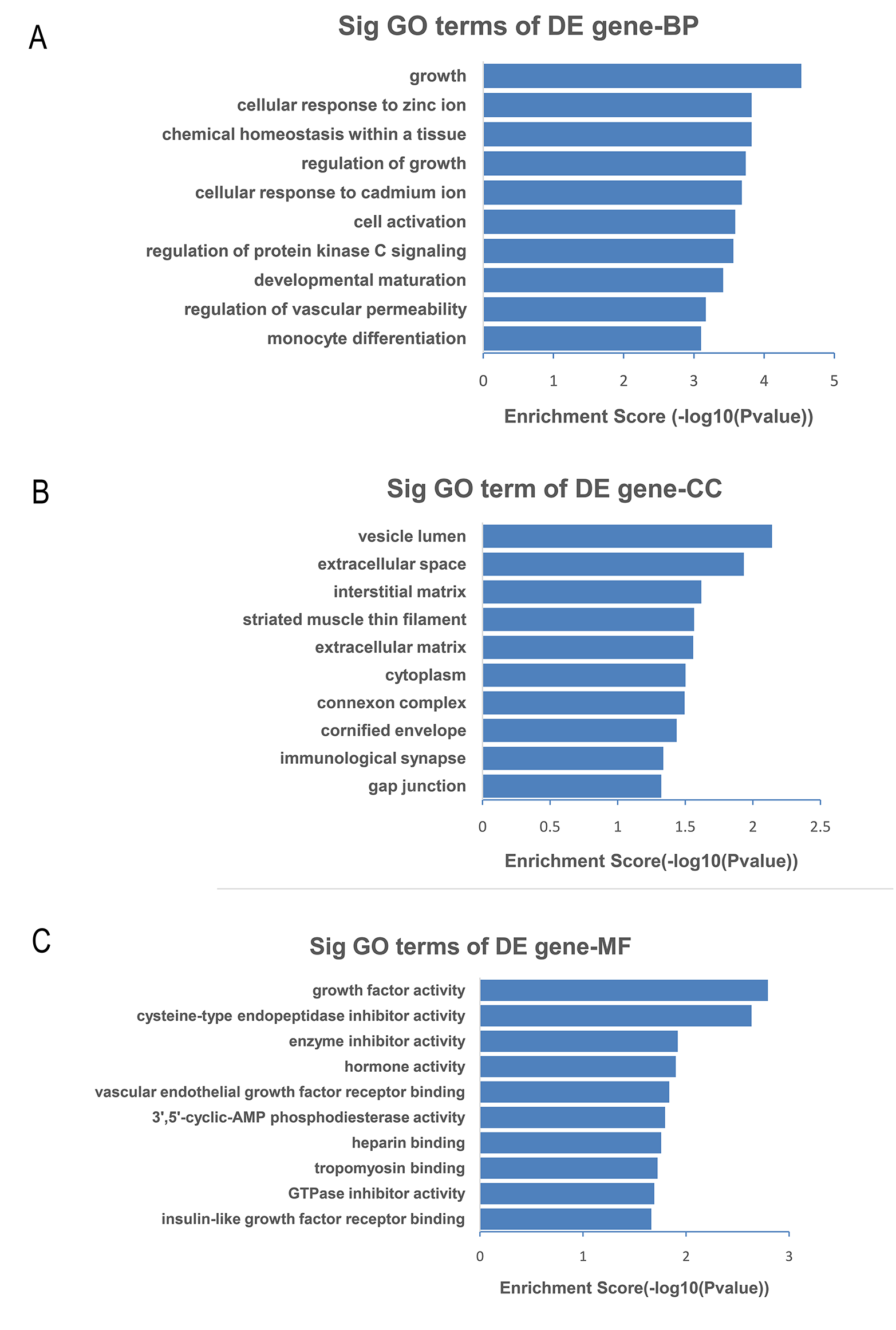

Supplement: Additional file 5: Figure S2. — Gene ontology (GO) analysis for up-regulated mRNAs. a Biological process (BP) analysis for up-regulated mRNAs. b Cellular component (CC) analysis for up-regulated mRNAs. c Molecular function (MF) analysis for up-regulated mRNAs. (TIF 432 kb) [file 13075_2016_1129_MOESM5_ESM.tif]

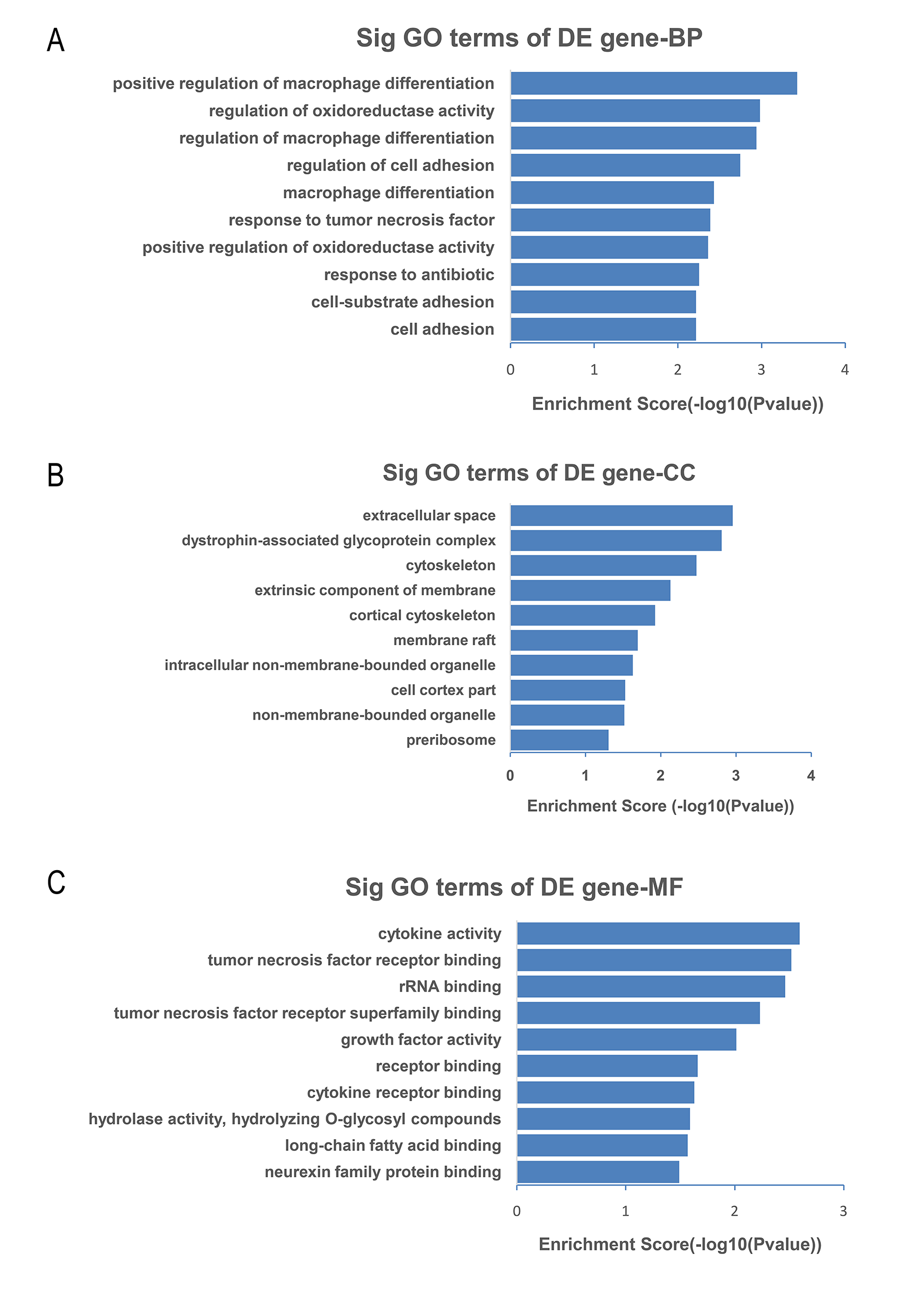

Supplement: Additional file 6: Figure S3. — Gene ontology (GO) analysis for down-regulated mRNAs. a Biological process (BP) analysis for down-regulated mRNAs. b Cellular component (CC) analysis for down-regulated mRNAs. c Molecular function (MF) analysis for down-regulated mRNAs. (TIF 468 kb) [file 13075_2016_1129_MOESM6_ESM.tif]

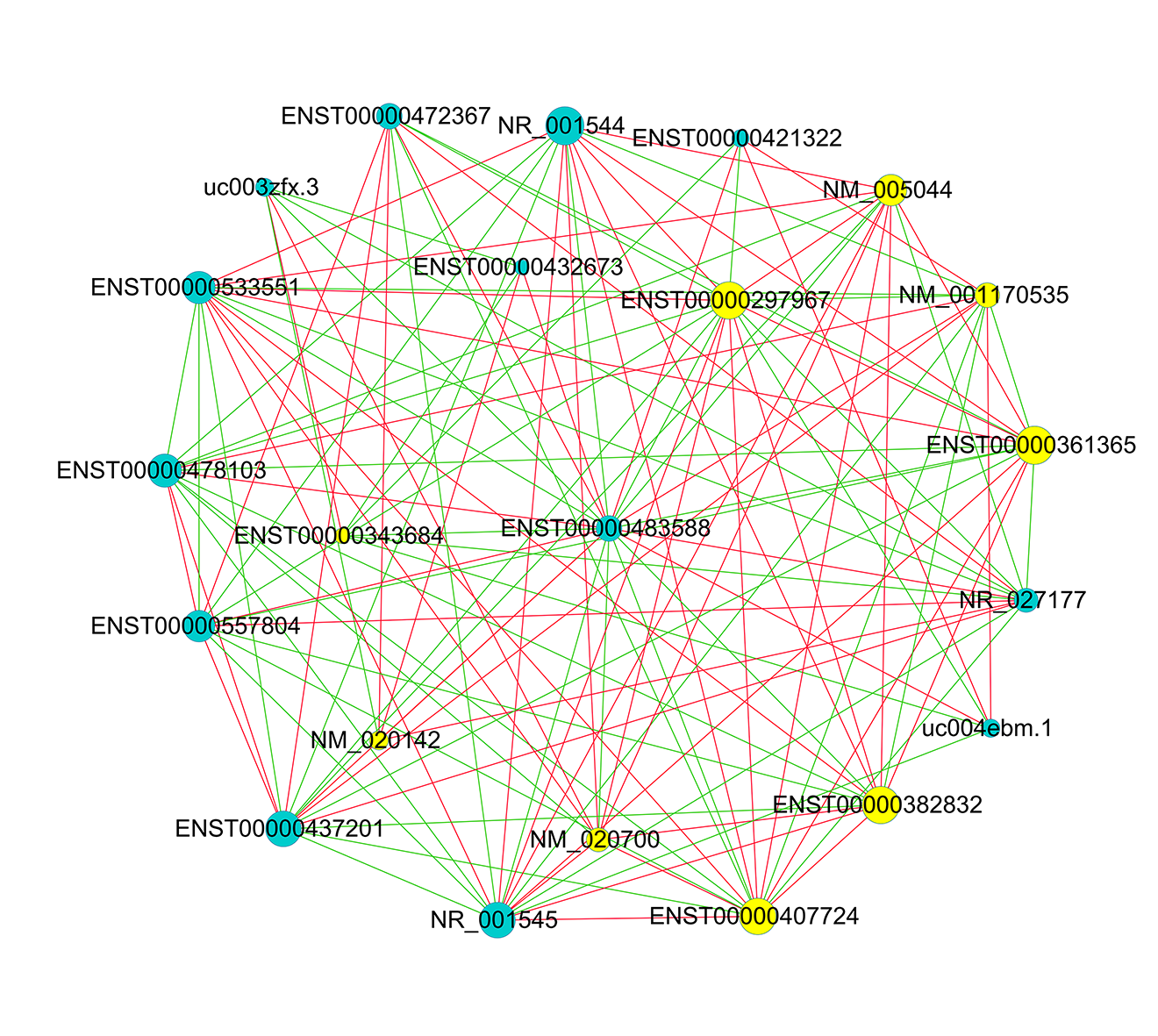

Supplement: Additional file 7: Figure S4. — Co-expression network of the differentially expressed lncRNAs and mRNAs. ENST00000483588 was connected to 12 lncRNAs and 9 mRNAs. Blue nodes represent lncRNAs and yellow nodes represent protein-coding genes. A red line represents a positive correlation, and a green line represents negative correlation. (TIF 863 kb) [file 13075_2016_1129_MOESM7_ESM.tif]

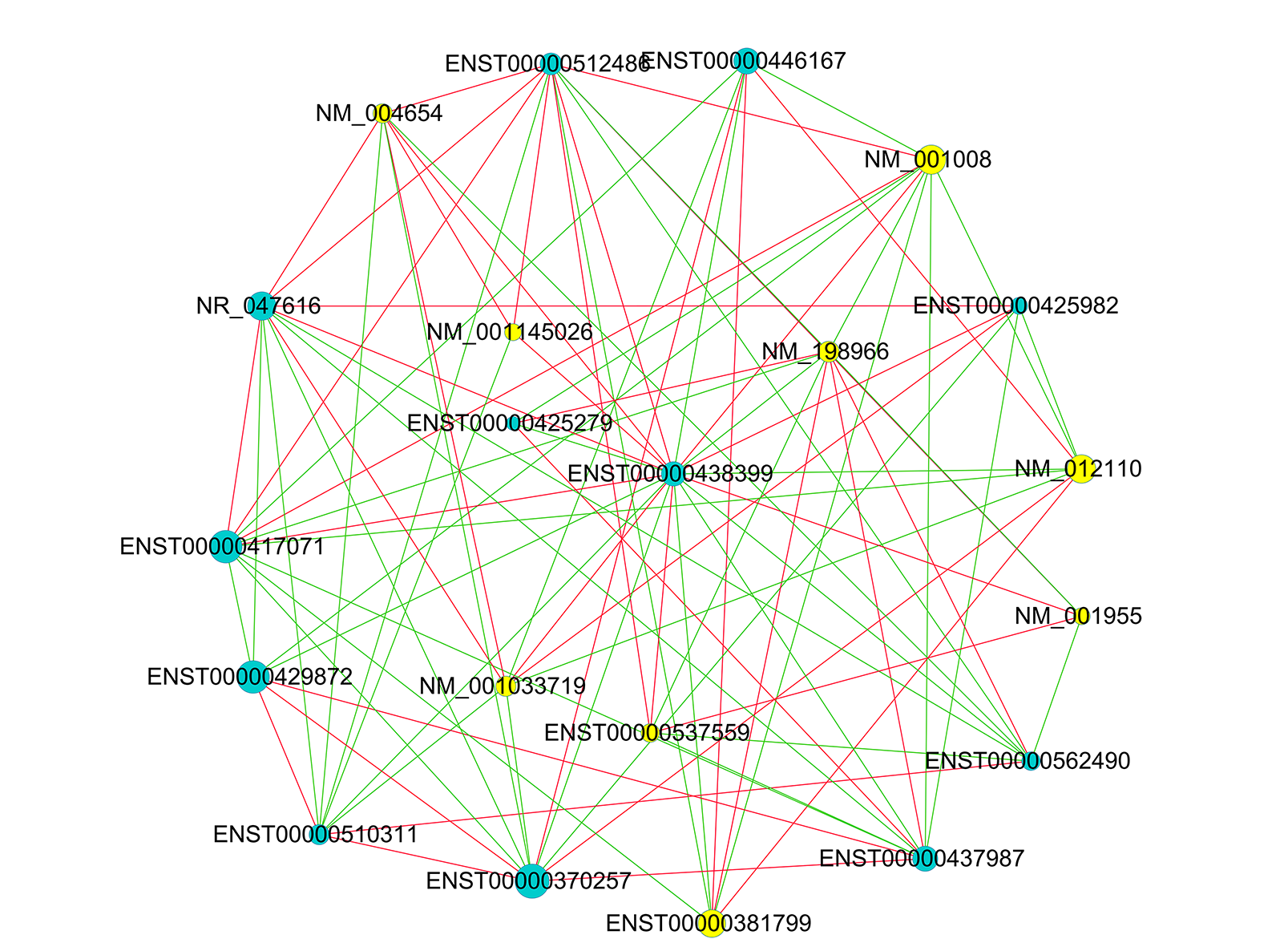

Supplement: Additional file 8: Figure S5. — Co-expression network of the differentially expressed lncRNAs and mRNAs. ENST00000438399 was connected to 11 lncRNAs and 9 mRNAs. Blue nodes represent lncRNAs and yellow nodes represent protein-coding genes. A red line represents positive correlation, and a green line represents negative correlation. (TIF 756 kb) [file 13075_2016_1129_MOESM8_ESM.tif]

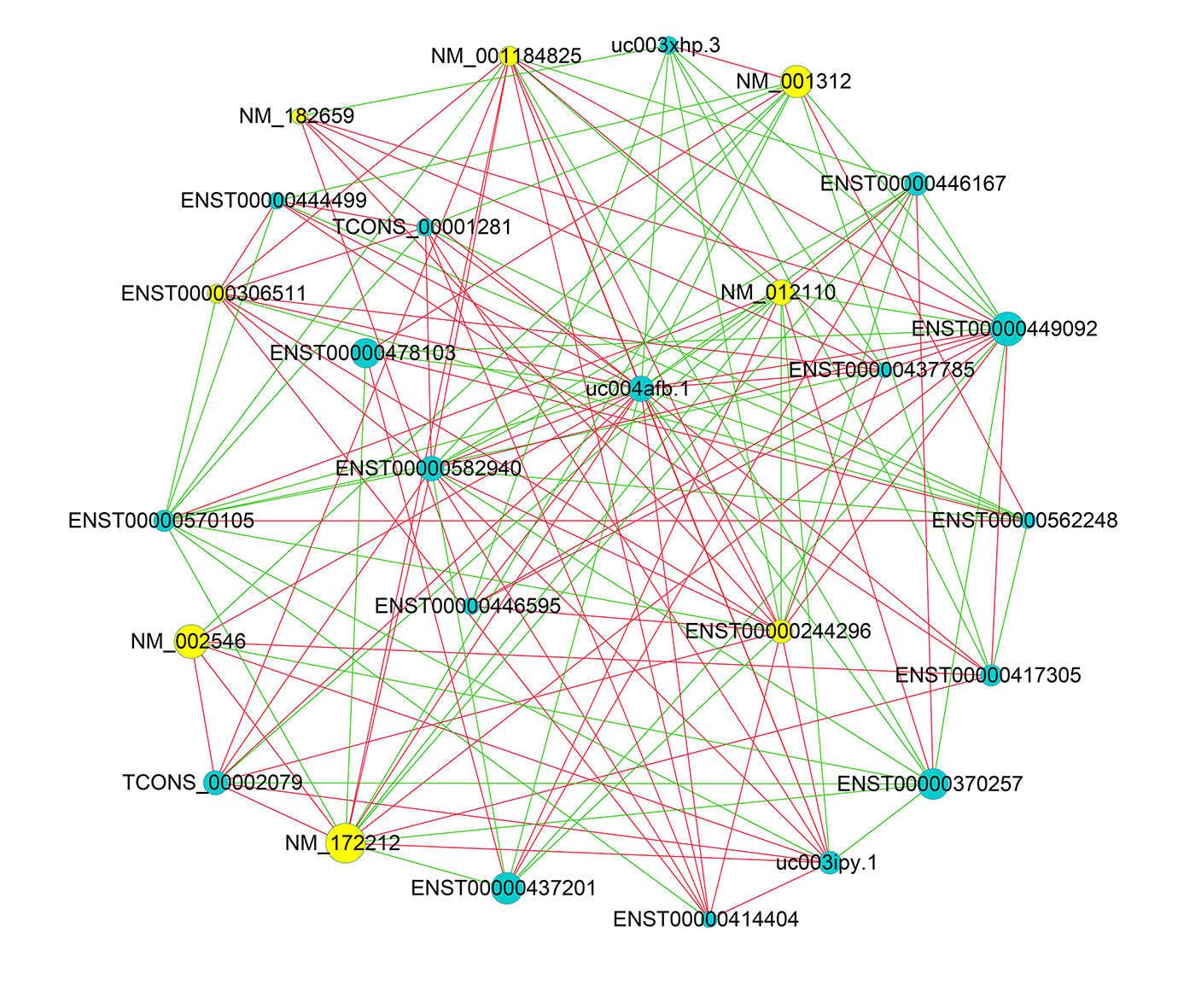

Supplement: Additional file 9: Figure S6. — Co-expression network of the differentially expressed lncRNAs and mRNAs. uc004afb.1 was connected to 17 lncRNAs and 8 mRNAs. Blue nodes represent lncRNAs and yellow nodes represent protein-coding genes. A red line represents positive correlation, and a green line represents negative correlation. (TIF 889 kb) [file 13075_2016_1129_MOESM9_ESM.tif]

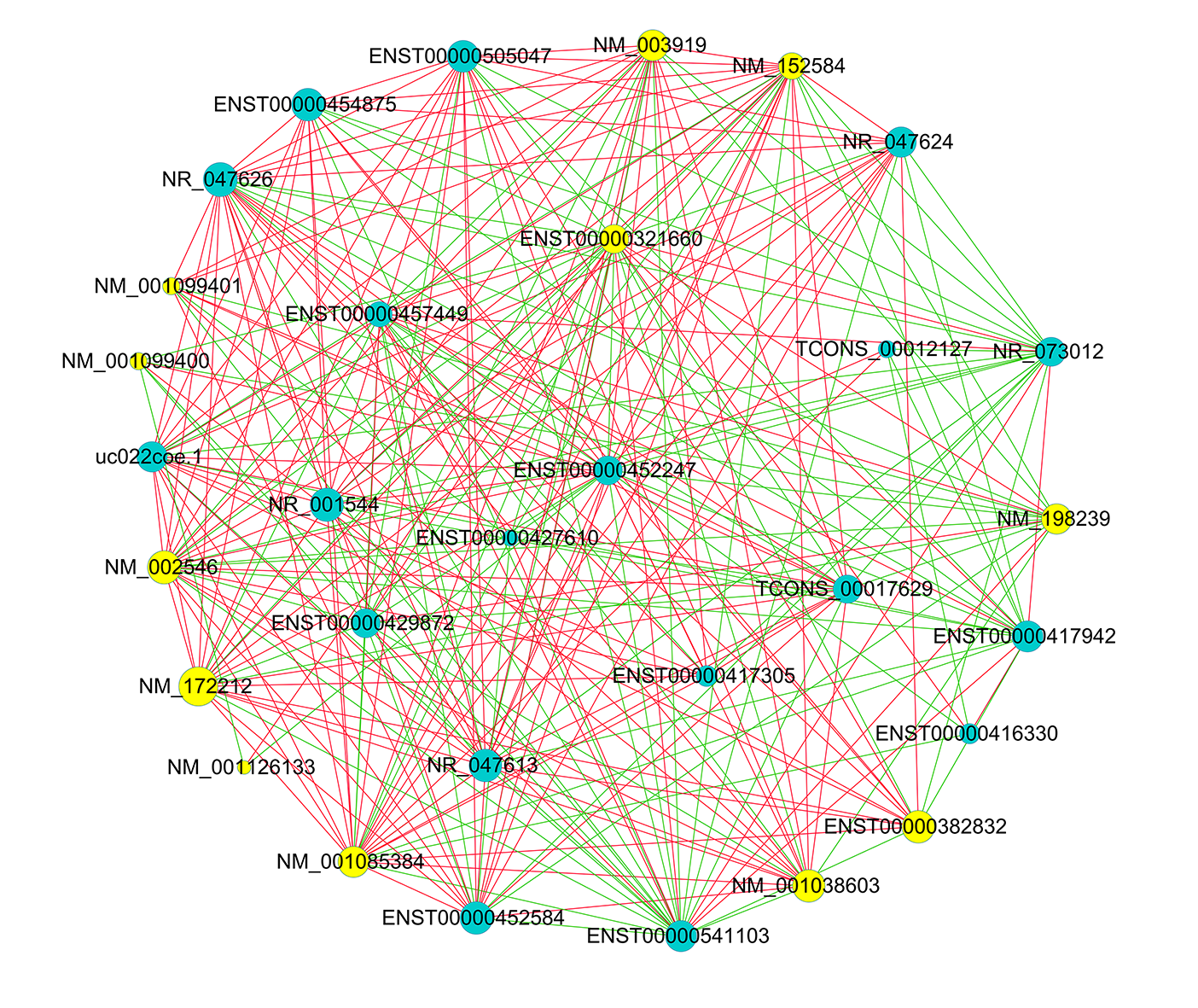

Supplement: Additional file 10: Figure S7. — Co-expression network of the differentially expressed lncRNAs and mRNAs. ENST00000452247 was connected to 18 lncRNAs and 12 mRNAs. Blue nodes represent lncRNAs and yellow nodes represent protein-coding genes. A red line represents positive correlation, and a green line represents negative correlation. (TIF 1529 kb) [file 13075_2016_1129_MOESM10_ESM.tif]

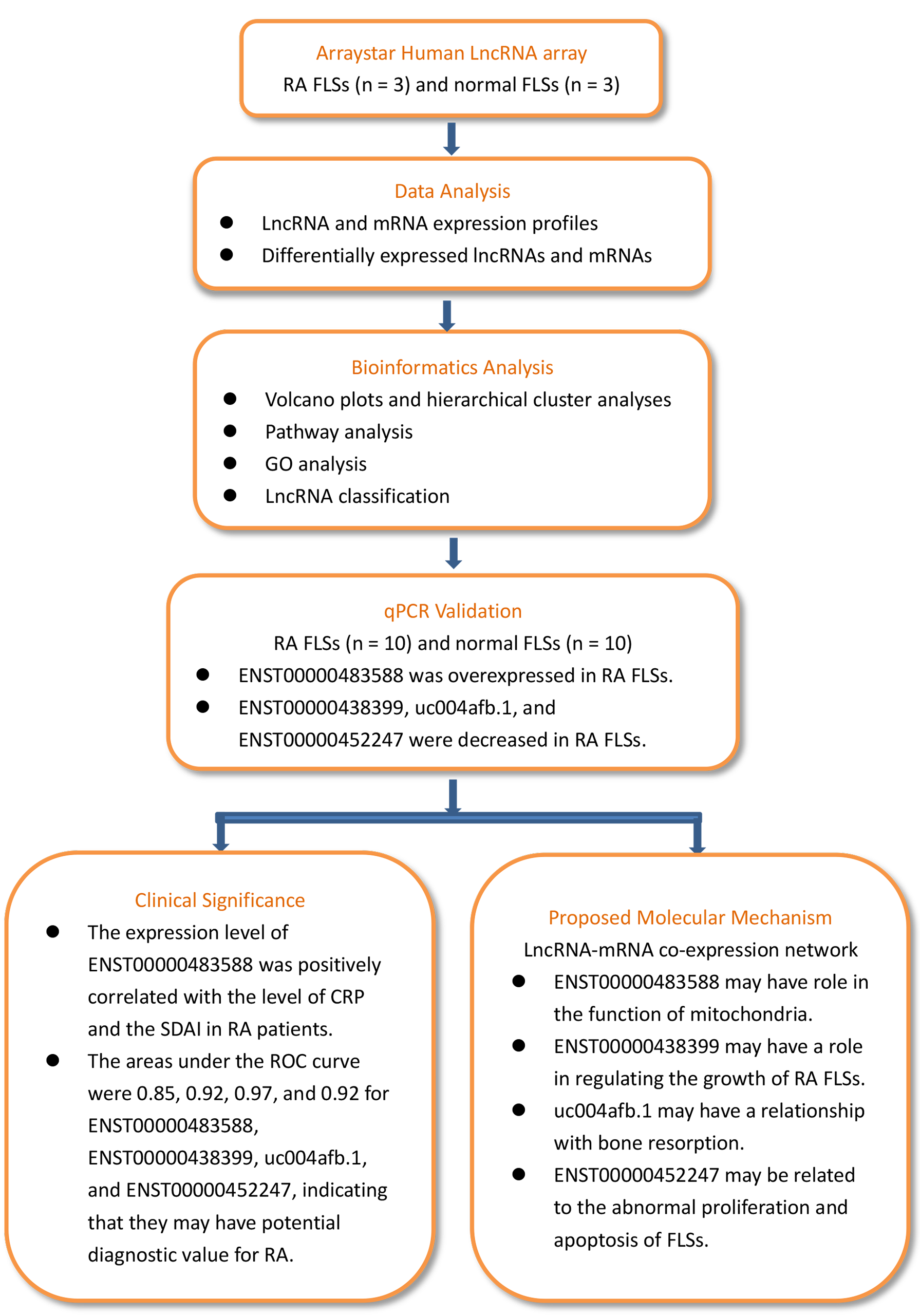

Supplement: Additional file 11: Figure S8. — Schematic diagram of the study design and summary of main findings. (TIF 814 kb) [file 13075_2016_1129_MOESM11_ESM.tif]
